# Plasma Lysophosphatidylcholine Levels Are Reduced in Obesity and Type 2 Diabetes

**DOI:** 10.1371/journal.pone.0041456

**Published:** 2012-07-25

**Authors:** Melissa N. Barber, Steve Risis, Christine Yang, Peter J. Meikle, Margaret Staples, Mark A. Febbraio, Clinton R. Bruce

**Affiliations:** 1 Biobank, Baker IDI Heart and Diabetes Institute, Melbourne, Victoria, Australia; 2 Cellular and Molecular Metabolism Laboratory, Baker IDI Heart and Diabetes Institute, Melbourne, Victoria, Australia; 3 Metabolomics Laboratory, Baker IDI Heart and Diabetes Institute, Melbourne, Victoria, Australia; 4 Department of Epidemiology, Monash University, Melbourne, Victoria, Australia; 5 Department of Physiology, Monash University, Melbourne, Victoria, Australia; The University of Tokyo, Japan

## Abstract

**Background:**

Obesity and type 2 diabetes (T2DM) are associated with increased circulating free fatty acids and triacylglycerols. However, very little is known about specific molecular lipid species associated with these diseases. In order to gain further insight into this, we performed plasma lipidomic analysis in a rodent model of obesity and insulin resistance as well as in lean, obese and obese individuals with T2DM.

**Methodology/Principal Findings:**

Lipidomic analysis using liquid chromatography coupled to mass spectrometry revealed marked changes in the plasma of 12 week high fat fed mice. Although a number of triacylglycerol and diacylglycerol species were elevated along with of a number of sphingolipids, a particularly interesting finding was the high fat diet (HFD)-induced reduction in lysophosphatidylcholine (LPC) levels. As liver, skeletal muscle and adipose tissue play an important role in metabolism, we next determined whether the HFD altered LPCs in these tissues. In contrast to our findings in plasma, only very modest changes in tissue LPCs were noted. To determine when the change in plasma LPCs occurred in response to the HFD, mice were studied after 1, 3 and 6 weeks of HFD. The HFD caused rapid alterations in plasma LPCs with most changes occurring within the first week. Consistent with our rodent model, data from our small human cohort showed a reduction in a number of LPC species in obese and obese individuals with T2DM. Interestingly, no differences were found between the obese otherwise healthy individuals and the obese T2DM patients.

**Conclusion:**

Irrespective of species, our lipidomic profiling revealed a generalized decrease in circulating LPC species in states of obesity. Moreover, our data indicate that diet and adiposity, rather than insulin resistance or diabetes *per se*, play an important role in altering the plasma LPC profile.

## Introduction

Obesity is an escalating health problem that is associated with chronic diseases including insulin resistance and type 2 diabetes mellitus (T2DM). Although the precise mechanism linking obesity to insulin resistance and T2DM are unknown, extensive evidence suggests that dyslipidemia plays a central role [1]. This is characterised by an increase in plasma free fatty acids (FFA) and triacylglycerols (TG) [2], [3]. Nonetheless, it is possible that obesity may impact upon nearly all indices of lipid homeostasis and that other circulating lipids contribute to the development of insulin resistance. Little is known about the plasma lipidome in both human obesity and rodent models of obesity and insulin resistance. With the introduction of high throughput mass spectometery-based technologies, it is now possible to perform comprehensive lipidomic analysis in body fluids such as plasma. Applying this novel technology to study circulating lipid changes in obesity may provide valuable information to help elucidate the mechanisms responsible for the development of T2DM.

To date, only a limited number of studies have examined the plasma lipidome in obesity [4]–[7]. The results of a lipidomic screen in monozygotic twins discordant for obesity showed that lysophosphatidylcholine (LPC) species were increased while ether phospholipids were decreased with obesity [6]. While, Graessler et al. [4] reported that obesity was associated with markedly increased levels of saturated TG and diacylglycerol (DG) species. Furthermore, diet-induced weight loss in obese individuals resulted in a reduction in saturated and short-chain fatty acid-containing TGs which correlated with improved insulin sensitivity [5]. Interestingly, the level of LPCs was unaltered by weight loss [5]. While these data provide important information on the effect that obesity has on the plasma lipidome, it is not known whether the presence of T2DM leads to specific alterations in lipids independent of obesity. Although obesity is associated with T2DM, it is important to note that not all obese individuals develop T2DM [8]–[10]. Therefore, it is important to investigate whether T2DM is associated with specific changes in the plasma lipidome. Indeed, identification of these lipid species may help to elucidate the mechanisms responsible for the progression from obesity to insulin resistance and T2DM and may uncover biomarkers to identify obese individuals at risk of developing T2DM.

Investigating mechanisms involved in the progression from obesity to insulin resistance and T2DM is difficult in humans. An alternative is to use animal models to gain an insight into the aetiology of human obesity and insulin resistance. The most common rodent model is the chronically high-fat fed mouse. Although routinely used, there is surprisingly little information regarding the plasma lipidome in this model. In this study, we performed lipidomic profiling of plasma from mice fed a high fat diet for 12 week. Further, to examine the temporal changes that occur in the progression from mild obesity to severe obesity and insulin resistance, we also performed time course studies in this model. Our rodent studies revealed that plasma LPCs were reduced with high fat-feeding. To establish the relevance of these findings to human obesity and T2DM, we performed analysis of plasma LPCs from lean, obese and obese T2DM individuals.

## Results

### Metabolic and Lipidomic Profile of Chronically Fat-fed Mice

**Figure 1:**
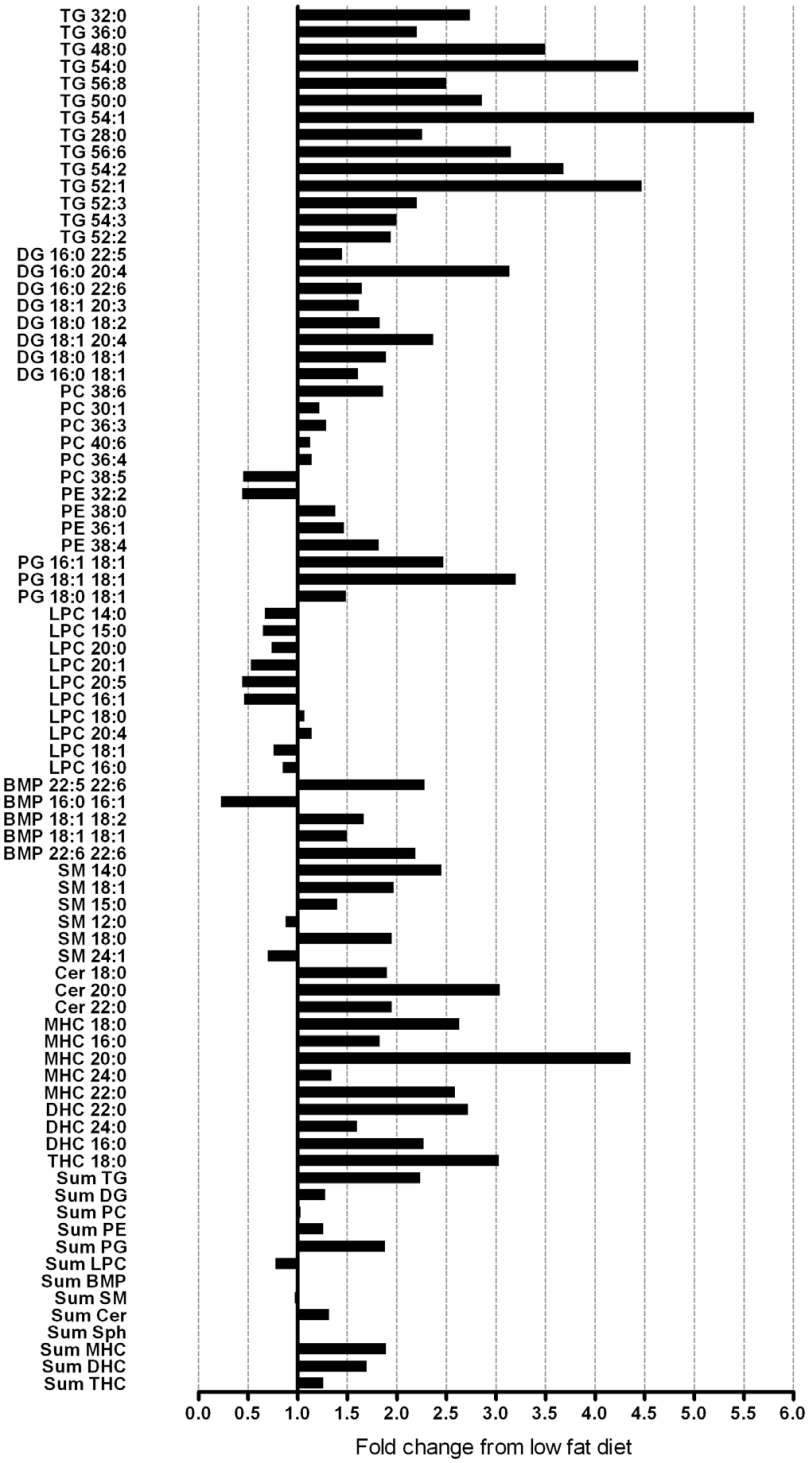
The effect of high-fat diet on the plasma lipidome. Significant mean fold changes (P<0.05) observed in plasma lipids from mice fed a high fat diet for 12 week (N = 6) relative to low fat fed animals (N = 8). Plasma lipids were analysed by LC ESI-MS/MS and absolute lipid levels were expressed relative to the low fat fed control group. Plasma samples were obtained from 5 hour fasted animals. BMP, bismonoacylglycerolphosphate; Cer, ceramide; DG, diacylglycerol; DHC, dihexosylceramide; LPC, lysophosphatidylcholine; MHC, monohexosylceramide; PC, phosphatidylcholine; PE, phosphatidylethanolamine; PG, phosphatidylglycerol; SM, sphingomyelin; Sph, sphingosine; TG, triacylglycerol; THC, trihexosylceramide.

**Figure 2:**
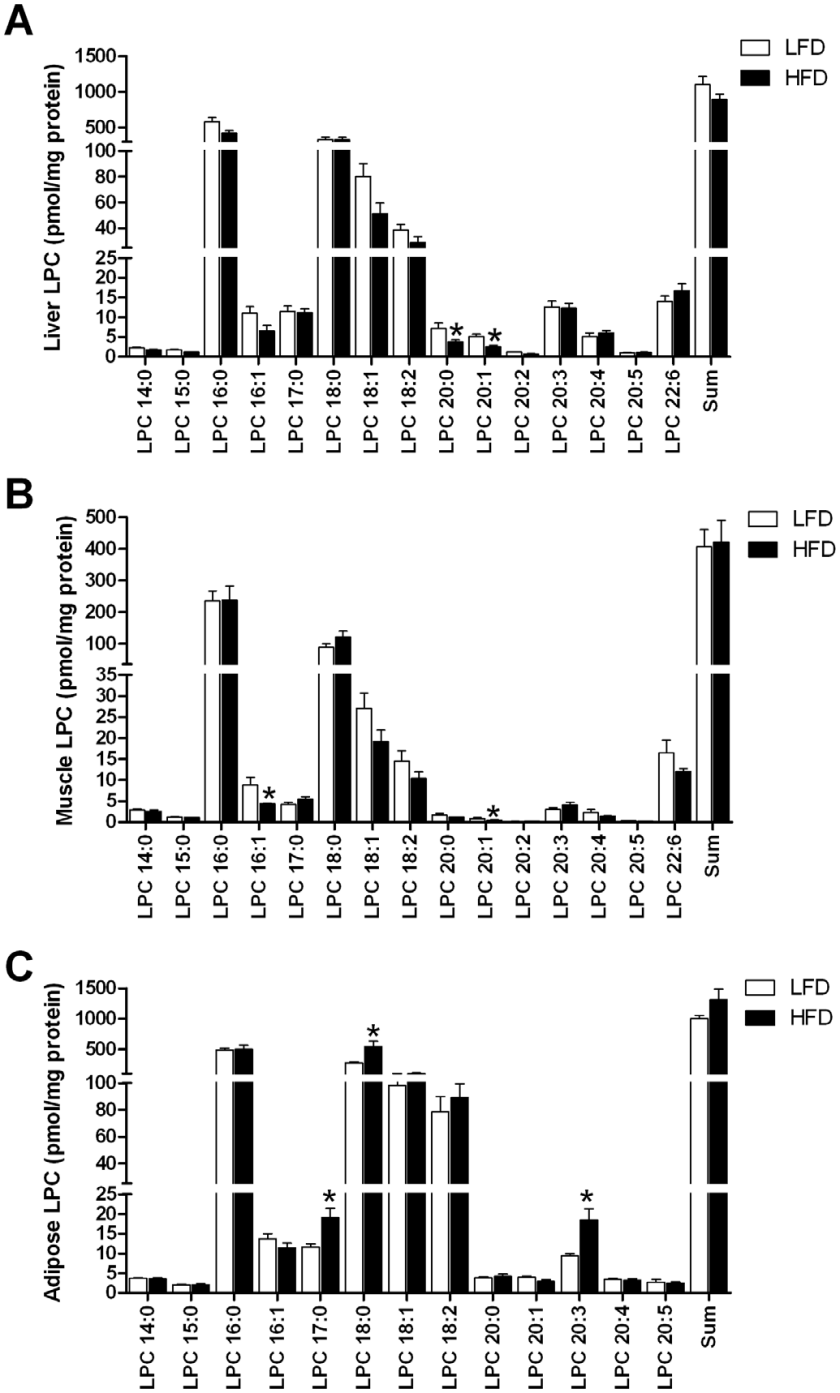
Tissue lysophosphatidylcholine levels in low and high fat fed mice. Liver (A), skeletal muscle (B) and adipose tissue (C) lysophosphatidylcholine (LPC) levels measured by LC ESI-MS/MS in mice fed a low (N = 8) or high fat diet (HFD, N = 6). Tissues were collected from 5 hour fasted animals. Data are presented as mean ± SEM. *P<0.05 versus low fat.

The high fat diet (HFD) increased body and fat mass (P<0.001; Table 1). Fat-fed mice displayed higher 5 hour fasted blood glucose (P<0.05) and plasma insulin levels (P<0.001) despite no change in plasma FFAs. As expected, a number of TG species were elevated in fat-fed mice (Figure 1). In fact of all lipid species analysed (Table S1), the largest increases were found in saturated TG species (3.5–5.5-fold increase) which on average had less than one double bond per fatty acid moiety (Figure 1). Notable increases were also found in DG species (Figure 1). In particular, DG that contained either palmitic (16∶0), stearic (18∶0) and oleic acid (18∶1) were elevated. Sphingomyelin, ceramide and hexoyslceramide levels were also increased with HFD (Figure 1). The HFD increased a number of phospholipid species, especially the PG and BMP species (Figure 1). Of particular interest was the finding of a generalised reduction in LPCs in the plasma of fat-fed mice (Figure 1). A number of studies have recently identified a role for LPCs in regulating glucose metabolism in both a positive and negative manner [11], [12], our subsequent studies therefore focused on LPCs. As liver, skeletal muscle and adipose tissue play vital roles in regulating glucose homeostasis and accumulation of bioactive lipids in these tissues is associated with insulin resistance, we next determined whether the HFD altered tissue LPC levels. In liver, the HFD resulted in a significant reduction in the levels of LPC 20∶0 and LPC 20∶1 (Figure 2A). Similarly, only modest changes in the muscle LPC profile was found with decreases in LPC 16∶0 and 20∶1 in response to fat feeding (Figure 2B). In contrast to the observations in liver and muscle, the levels of LPC 17∶0, 18∶0 and 20∶3 were elevated in adipose tissue of mice maintained on the HFD (Figure 2C). Despite the changes in molecular species in these tissues, the HFD failed to alter total LPC levels (Figure 2A–C). Thus, in contrast to our findings in plasma, only modest changes in tissue LPCs were noted.

**Table 1:** Metabolic parameters of C57Bl/6 mice fed a low or high fat diet for 12 wk.

	LFD	HFD
Body mass (g)	30.3±1.2	38.1±1.0^b^
Lean mass (g)	25.6±1.0	25.1±0.5
Fat mass (g)	3.9±0.4	12.1±0.8^b^
% lean mass	84.5±1.0	66.0±1.6^b^
% fat mass	13.0±1.4	31.6±1.5
Epididymal fat pad (g)	0.63±0.04	1.97±0.10^b^
Subcutaneous fat pad (g)	0.42±0.04	1.58±0.13^b^
Blood glucose (mM)	8.5±0.4	9.4±0.5^a^
Plasma insulin (pM)	62.1±10.7	401.0±94.0^b^
HOMA-IR	3.0±0.5	22.5±5.5^a^
Plasma free fatty acids (mM)	0.66±0.08	0.62±0.06
Plasma triglycerides (mM)	0.80±0.10	0.97±0.07

All measurements were made in 5 hour fasted mice. Data are mean ± SEM.

a P<0.05 vs Chow;

b P<0.001 vs Chow. LFD, low fat diet; HFD, high fat diet.

### Time Course Changes in the Metabolic Parameters and Plasma LPC Levels during Chronic Fat Feeding

**Figure 3:**
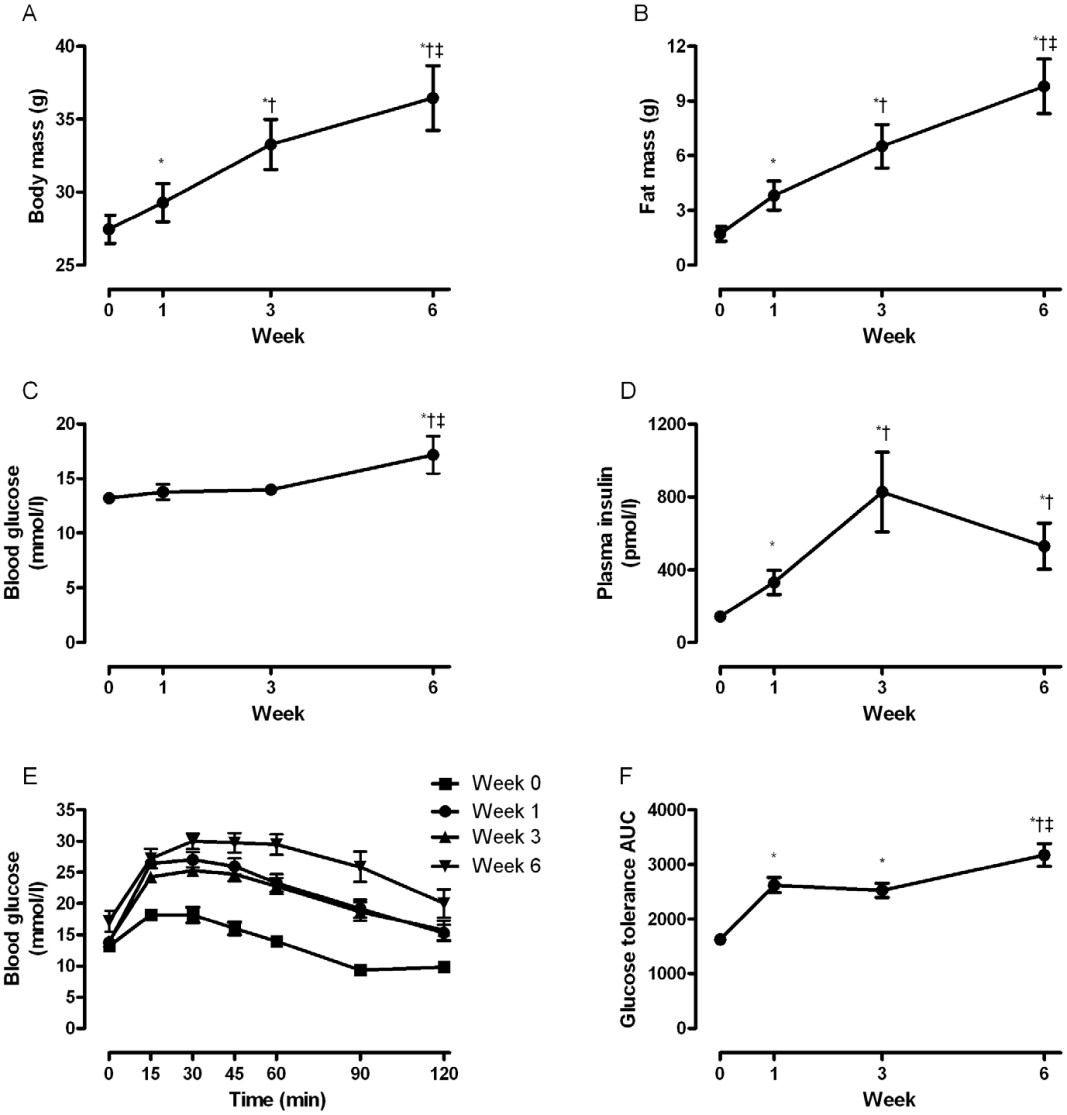
Time course changes in metabolic parameters measured in mice fed a high fat diet. Body mass (A), fat mass (B), fasting blood glucose (C), plasma insulin (D), blood glucose during an intraperitoneal glucose tolerance test (E) and blood glucose area under the curve during an intraperitoneal glucose tolerance test (F) were measured in mice (N = 11) following a 5 hour fast at baseline (time 0) and after 1, 3 and 6 weeks of high fat feeding. Data are presented as mean ± SEM. *P<0.05 versus baseline; ^†^P<0.05 versus week 1; ^‡^P<0.05 versus week 3.

In order to determine when the change in plasma LPCs occurred in response to the HFD, we performed time course studies in mice fed a HFD for 1, 3 and 6 weeks. Metabolic phenotyping was carried out at each of these time points. Body weight and fat mass were increased after only 1 week of HFD and were further elevated at 3 and 6 weeks (Figure 3A & B). Energy intake was increased in mice fed the HFD at week 1 (44.2±0.4 kJ/d for low-fat diet (LFD) vs 70.8±2.6 kJ/d for HFD, P<0.05) and week 3 (48.5±0.1 kJ/d for LFD vs 91.7±0.1 kJ/d for HFD, P<0.05). However, at week 6 energy intake was not different (62.0±4.8 kJ/d for LFD vs 57.3±0.3 kJ/d for HFD). Lean mass was not affected by the HFD (data not shown). Five hour fasted blood glucose was only elevated after 6 weeks of HFD (Figure 3C) while plasma insulin levels doubled after 1 week and was further increased at 3 weeks (Figure 3D). However, after 6 weeks of HFD, insulin levels decreased and were comparable to those following 1 week of HFD (Figure 3D). Whole body glucose intolerance developed after 1 week of HFD (Figure 3E & F), was unaltered between weeks 1 and 3 and after 6 weeks of HFD, was worse than that observed at either week 1 or 3 (Figure 3E & F).

**Figure 4:**
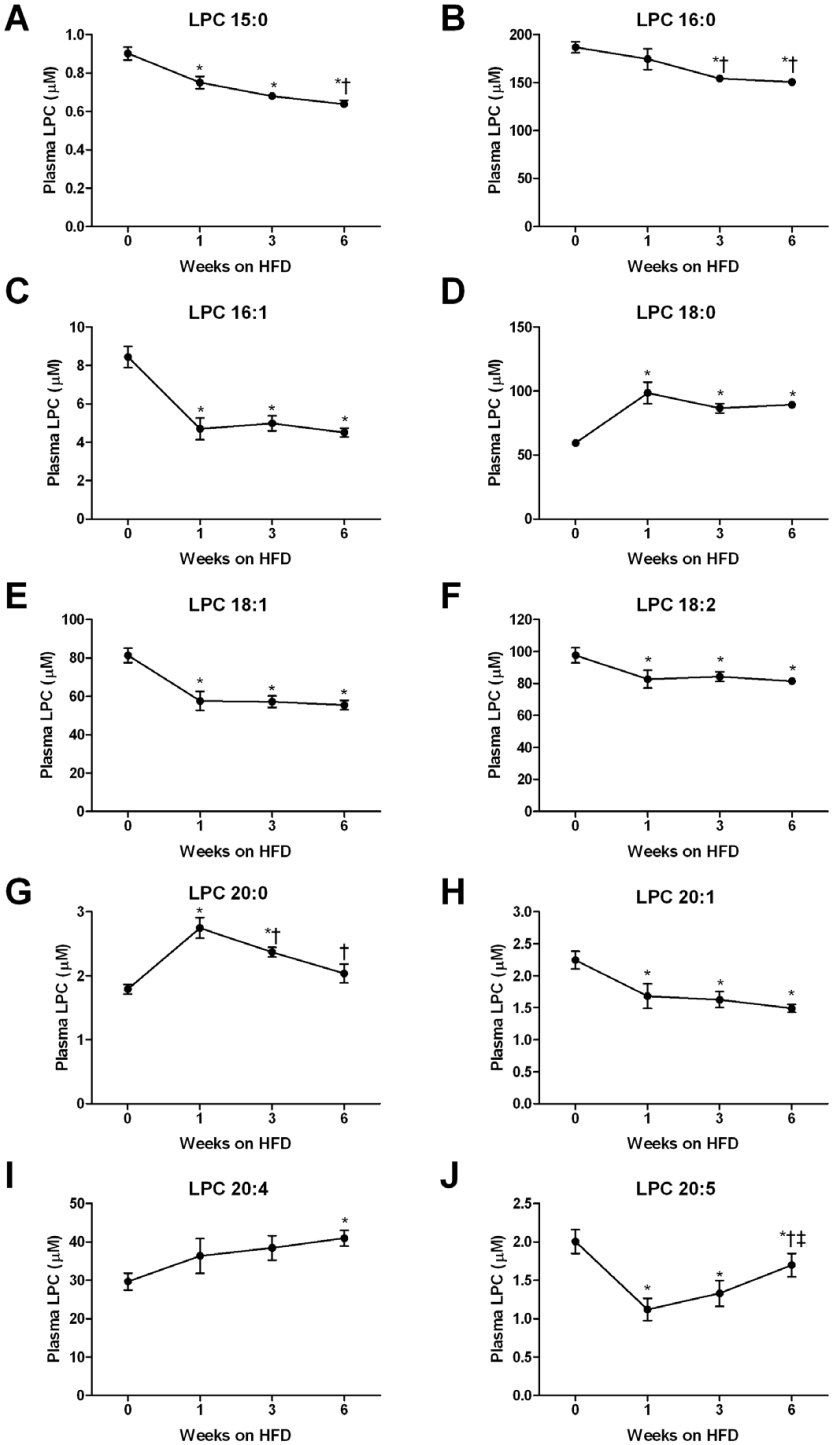
Time course changes in selected lysophosphatidylcholine (LPC) species measured in plasma of mice fed a high fat diet. LPC 15∶0 (A), LPC 16∶0 (B), LPC 16∶1 (C), LPC 18∶0 (D), LPC 18∶1 (E), LPC 18∶2 (F), LPC 20∶0 (G), LPC 20∶1 (H), LPC 20∶4 (I) and LPC 20∶5 (J) were measured in mice (N = 11) following a 5 hour fast at baseline (time 0) and after 1, 3 and 6 weeks of high fat feeding using LC ESI-MS/MS. Data are presented as mean ± SEM. *P<0.05 versus baseline; ^†^P<0.05 versus week 1; ^‡^P<0.05 versus week 3.

The plasma LPCs that were significantly altered over the HFD time course are presented in Figure 4. Interestingly, the HFD resulted in rapid alterations in the plasma LPC profile. Indeed, when compared with the profile of 12 week fat fed mice, over 80% of the changes noted at this point were evident after only 1 week of HFD (Figure 4). Importantly, the direction of change in the individual plasma LPC species was very similar to our 12 week HFD data. Specifically, rapid and sustained decreases in LPC 15∶0 (Figure 4A), 16∶1 (Figure 4C), 18∶1 (Figure 4E), 18∶2 (Figure 4F), 20∶1 (Figure 4H) and 20∶5 (Figure 4J) were detected in fat fed mice, while the decline in LPC 16∶0 (Figure 4B) occurred later in response the HFD. While the HFD caused a reduction in many LPC species, the saturated species LPC 18∶0 (Figure 4D) and 20∶0 (Figure 4G) were increased after 1 week of HFD, whereas the increase in LPC 20∶4 (Figure 4I) was only detected after 6 weeks. The changes in each lipid class analysed are presented in Table S2.

### Relationship between Circulating LPC Levels, Insulin Resistance and Adiposity

**Figure 5:**
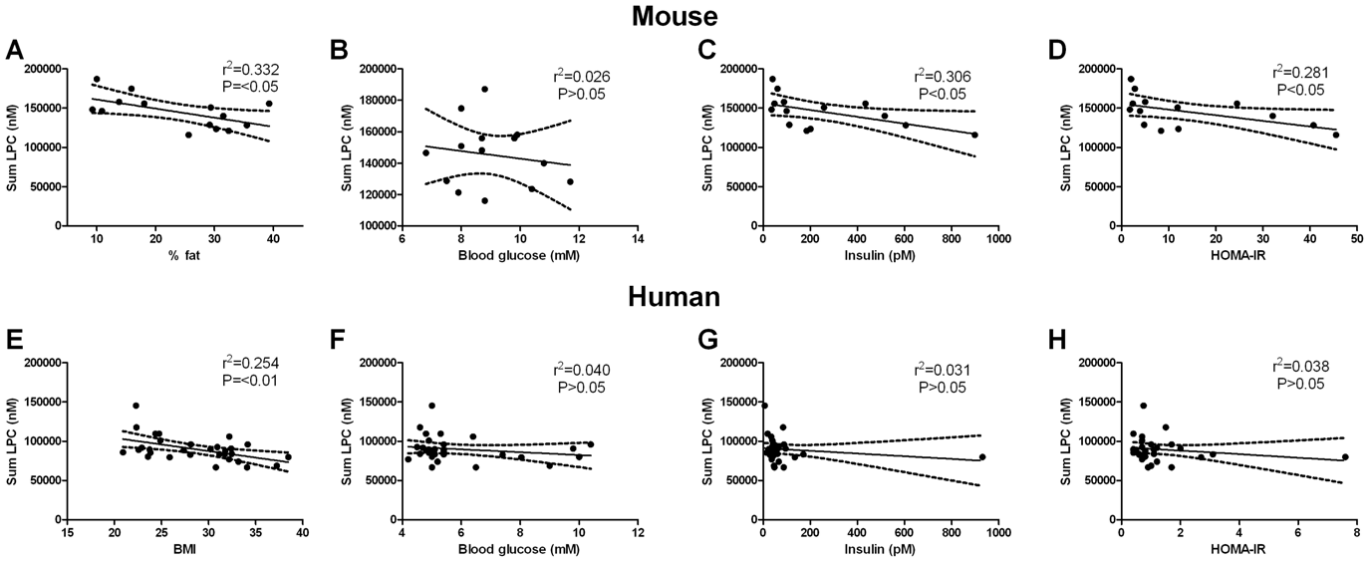
Relationship between plasma lysophosphatidylcholine (LPC), adiposity and indices of insulin resistance in high fat fed mice (A–D) and lean, obese and obese type 2 diabetic humans (E–H). The association between circulating LPC and percent body fat (A), blood glucose (B), plasma insulin (C) and HOMA-IR (D) in high fat fed mice. The association between circulating LPC and BMI (E), blood glucose (F), plasma insulin (G) and HOMA-IR (H) in the human cohort. Data are presented as line of best fit with 95% confidence intervals.

To examine the relationship between plasma LPC levels, insulin resistance (HOMA-IR), adiposity and diet, regression analysis was performed (Figure 5A–D and Tables S3, S4, S5). The regression analysis revealed that only three plasma LPC species (LPC 16∶1, LPC 20∶0 and LPC 20∶1) showed any significant association with HOMA-IR. However, when diet was included in the model these associations were lost. We also observed significant associations between relative adiposity (ie. % body fat) and circulating LPCs (Figure 5A–D). Again, when diet was included in the model these associations disappeared (Tables S3, S4, S5).

### Clinical Characteristics of the Human Cohort

The clinical characteristics of all subjects are shown in Table 2. The obese T2DM group was older than both the lean and obese non-diabetic groups (P<0.05; Table 2). The obese and obese T2DM groups were matched for body mass and BMI (Table 2) and both these groups had significantly greater body mass and BMI compared with the lean group (P<0.05) as intended (Table 2). Although blood glucose was higher in the obese T2DM group, no differences were noted in plasma insulin or HOMA-IR (Table 2). In addition, no differences were found between groups for plasma TGs, FFA, total, LDL or HDL cholesterol and blood pressure (Table 2).

**Table 2:** Subject characteristics.

	Lean (N = 11)	Obese non-diabetic (N = 10)	Obese type 2 diabetic (N = 9)
Age (years)	51±2	49±3	60±2a,b
Height (m)	1.78±0.02	1.78±0.02	1.74±0.02
Body mass (kg)	74.5±2.1	101.2±3.9^a^	95.5±4.7^a^
BMI	23.3±0.4	31.9±0.4^a^	31.5±1.4^a^
Blood glucose (mM)	5.0±0.1	4.9±0.1	8.0±0.6a,b
Plasma insulin (pM)	36.1±1.1	60.4±13.9	157.6±92.4
HOMA-IR	0.7±0.1	1.1±0.2	2.0±0.7
Plasma free fatty acids (mM)	0.28±0.05	0.22±0.03	0.33±0.05
Plasma triglycerides (mM)	1.0±0.3	1.4±0.2	1.5±0.2
Plasma total cholesterol (mM)	4.6±0.2	4.6±0.2	5.0±0.3
Plasma LDL (mM)	2.9±0.3	2.8±0.2	2.8±0.4
Plasma HDL (mM)	1.2±0.1	1.2±0.1	1.6±0.2
Systolic blood pressure (mmHg)	119±2	122±2	128±4
Diastolic blood pressure (mmHg)	78±2	78±2	78±2

Samples were obtained following an overnight (10–12 hour) fast. Data are mean ± SEM.

a P<0.05 vs lean;

b P<0.05 vs obese non-diabetic.

### Changes in Plasma LPCs in Human Obesity and T2DM

Consistent with our mouse model of obesity, a large number of LPC species, as well as the total LPC levels, were reduced in the plasma of obese healthy and obese T2DM groups when compared with lean individuals (Table 3). Adjustment for age and common clinical indices of disease did not affect the associations between these lipids and disease status. Interestingly, we did not find any differences between the obese and obese T2DM groups even when direct comparisons were made, suggesting that obesity *per se* may play a role in modulating plasma LPC levels. To explore this possibility, we examined the relationship between LPC species and indices of metabolic homeostasis (i.e. body mass, BMI, blood glucose, plasma insulin, FFA, TGs, total, LDL and HDL cholesterol). This analysis did indeed reveal that plasma LPC species were negatively associated with body mass (data not shown) and BMI (Figure 5; Table S6). In addition, significant negative correlations were found between a number of LPC species and plasma insulin levels (Table S6). There was no relationship between plasma LPCs and other indices such as blood glucose, HOMA-IR and plasma lipids.

**Table 3:** Plasma LPC levels in lean, obese non-diabetic and obese type 2 diabetic individuals.

LPC species (µM)	Lean (N = 11)	Obese non-diabetic (N = 10)	Obese type 2 diabetic (N = 9)
LPC 14∶0	0.92±0.14	0.76±0.10	0.78±0.06
LPC 15∶0	0.56±0.03	0.46±0.03^a^	0.47±0.03^c^
LPC 16∶0	34.11±1.26	31.56±1.14	32.65±0.98
LPC 16∶1	2.57±0.61	1.97±0.21	2.26±0.40
LPC 18∶0	14.37±0.54	12.35±0.36^b^	11.96±0.56^b^
LPC 18∶1	15.91±1.85	11.63±0.58^a^	12.51±1.13
LPC 18∶2	21.57±1.40	16.25±0.99^b^	16.07±1.20^b^
LPC 20∶0	0.10±0.01	0.08±0.01^c^	0.06±0.01^b^
LPC 20∶1	0.20±0.03	0.13±0.01^d^	0.15±0.01^a^
LPC 20∶2	0.23±0.03	0.14±0.01	0.15±0.01^a^
LPC 20∶3	2.08±0.23	1.64±0.10	1.64±0.16
LPC 20∶4	5.45±0.39	4.60±0.28^a^	3.80±0.35^b^
LPC 20∶5	0.98±0.15	0.76±0.09	0.77±0.11
LPC 22∶6	1.50±0.16	1.18±0.11	1.15±0.18
Sum LPC	100.55±5.76	83.53±2.81^a^	84.42±4.17^a^

Samples were obtained following an overnight (10–12 hour) fast. Data are mean ± SEM.

a P<0.05 vs lean;

b P<0.01 vs lean;

c P = 0.052 vs lean;

d P = 0.06 vs lean.

## Discussion

Dyslipidemia plays an important role in the development of insulin resistance (1). Although this generally manifests as increases in circulating FFAs and TGs, it is likely that the content and composition of a number of other lipid species is altered and contributes to the pathogenesis of T2DM. In order to gain more insight into the changes that occur in circulating lipids in obesity, we performed lipidomic analysis on the chronically fat fed mouse, a well characterised and highly utilised rodent model of obesity and insulin resistance. Although we found that exposing mice to chronic HFD markedly altered the lipidomic profile, the most striking observation was the generalized reduction in plasma LPC species. Time course studies revealed that most of these changes in LPCs occurred within the first week of diet intervention and coincided with an increase in fat mass and the development of glucose intolerance and hyperinsulinemia. Consistent with our rodent model, human studies revealed a widespread reduction in LPC species in plasma from obese and obese T2DM individuals. Interestingly, we were unable to detect any differences when comparing obese individuals with the T2DM patients. Therefore, our data indicate that obesity-related factors, such as diet and adiposity, rather than insulin resistance and diabetes *per se*, may make the major contribution to the changes in LPC profile observed.

Animal models are routinely used to study obesity and insulin resistance, however, little is known about the plasma lipidome in these models. Our first aim was to examine the plasma lipid profile of mice fed a HFD for 12 weeks. Fat feeding resulted in marked changes in the plasma lipid profile (Figure 1) including increases in a number of TG and DG species. In particular, most of the DG species that were increased contained either palmitic (16∶0); steraic (18∶0) or oleic acid (18∶1) which reflects the fatty acid composition of our HFD. The HFD also caused an increase in a restricted subset of PC species, but these changes were minor compared with the magnitude of change in other lipids such as TG. In addition, the HFD resulted in specific increases in the saturated ceramide species 18∶0, 20∶0 and 22∶0. These findings are consistent with a previous report demonstrating that plasma ceramide levels are elevated in the obese, insulin resistant *ob/ob* mouse [13]. Furthermore, it has been reported that plasma ceramides are elevated in T2DM humans [14]. Despite this accumulating data demonstrating that circulating ceramides are elevated with obesity, there is no evidence to suggest that plasma ceramides have a mechanistic role in the development of insulin resistance.

The novel and unexpected finding of this study was the general trend for LPCs to be reduced in plasma from chronically fat-fed mice. LPC is an important signalling molecule with diverse biological functions and is involved in regulating cellular proliferation, tumour cell invasion and inflammation [15]–[18]. As obesity is associated with chronic low-grade inflammation [19], one may expect LPCs to be increased with obesity. Indeed, plasma LPCs have been reported to be elevated in obesity [6] and T2DM [20]. In addition, liver and skeletal muscle LPC levels are increased in the obese diabetic *db/db* mouse and lowering tissue LPCs ameliorated insulin resistance and diabetes in these mice [11]. Taken together, these findings support the recent notion that LPC may be involved in mediating insulin resistance in obesity [11]. However, our results contrast these findings. We clearly show that 12 weeks of fat feeding, which induces severe obesity, is associated with a reduction in plasma LPC levels. Our data are in support of recent studies demonstrating that plasma LPC levels are reduced in individuals with impaired glucose tolerance [21] and in a mouse model of steatohepatitis [22]. Thus, evidence is emerging to indicate that metabolic disorders are associated with a reduction in plasma LPCs. The mechanism responsible for the reduction in circulating LPC is unknown but may be due to an increase in breakdown or enhanced clearance from the circulation by metabolically active tissues. We therefore examined the LPC content of a number of metabolically active tissues (liver, muscle and adipose) in order to determine whether elevated uptake and storage of LPC may contribute not only to lower circulating LPC levels but also to the development of insulin resistance. In contrast to Han et al. [11], we failed to show that the HFD caused an increase in LPC content in liver and muscle, the two most important tissues in regulating insulin sensitivity. In fact, if anything, we show that the HFD decreased some molecular species of LPC in these tissues. Adipose tissue is also involved in regulating metabolism and is a major site of metabolic inflammation [19], [23], [24]; thus we examined LPC levels in this tissue. Consistent with our findings in liver and muscle, total LPC levels were unaltered in adipose tissue from fat fed mice. However, unlike liver and muscle, we detected increases in a very small number of LPC species. Whether this small increase in such a restricted subset of LPC species is able to alter the metabolic and inflammatory profile of adipose tissue and subsequent whole body insulin sensitivity is not known. Together with the fact that the inflammatory response initiated in adipose tissue is proposed to cause insulin resistance by stimulating lipolysis and increasing circulating FFAs [23] and that the HFD did not alter plasma FFAs, we speculate that alterations in the adipose LPC profile are unlikely to contribute to the development of whole body insulin resistance.

Recent findings also suggest that nutritional status can influence circulating LPC levels [25]. While Kus et al. [25] reported no differences in plasma LPCs in the fasted state, upon re-feeding a number of LPC species (18∶0, 18∶2 and 20∶4) were elevated in high fat fed mice. Although these findings may have implications for our study as we collected plasma in the fasted state (5 hour fast), the fasting conditions used by Kus et al. [25] were much longer and more extreme (ie. 14 hour fast) than used in the current study. Such an extended fast is likely to cause many changes that may alter metabolism including a reduction in body mass and depletion of liver glycogen content. Nonetheless, these data do provide evidence that nutritional status can influence circulating LPC levels and should be a factor taken into consideration when performing lipidomic profiling.

Our studies of chronically fat-fed mice clearly show that the presence of obesity and insulin resistance alter the plasma LPC levels. However, these results do not provide a great deal of insight into the temporal changes that occur in plasma LPCs in the development of obesity and insulin resistance. We therefore performed time course studies to examine when obesity and insulin resistance develop with the HFD and attempted to identify whether this is associated with alterations in the plasma LPC profile. After only one week of HFD, significant increases in adiposity were found together with a doubling in plasma insulin despite no change in blood glucose, indicative of the development of insulin resistance. In addition, mice developed glucose intolerance. This rapid induction of obesity, hyperinsulinemia and glucose intolerance was associated with alterations in the plasma LPC profile. In fact, of all LPC species that were altered over the 6 week HFD intervention, >80% of these changes were detected after the first week. An interesting point to note is that despite further increases in adiposity at 3 and 6 weeks of HFD and additional deterioration in glucose tolerance at week 6, the plasma LPC profile remained relatively unchanged after the first week. Furthermore, the actual changes in the LPC species largely reflected those alterations we observed with our initial 12 week fat feeding study.

Despite showing that HFD caused rapid alterations in the plasma LPC profile, we were unable to dissect out the exact role of obesity versus insulin resistance in contributing to these changes. At the earliest time point studied, mice had already gained significant fat mass which coincided with the development of glucose intolerance and hyperinsulinemia. Therefore, it is difficult to examine whether the high fat diet, obesity or insulin resistance *per se* is the major factor causing these changes in circulating LPC. To try to further define this relationship, regression analysis was performed. This revealed that only a limited number of LPC species (LPC 16∶1, LPC 20∶0 and LPC 20∶1) were associated with HOMA-IR. However, when diet was included in the model no associations were found between HOMA-IR and plasma LPC species suggesting that diet was more important than insulin resistance in determining circulating LPC levels. Furthermore, our analysis revealed that a number of LPC species were associated with adiposity (ie. % fat mass). Interestingly, these associations tended to disappear when diet was included in these models. Thus, while adiposity does appear to play a role in determining circulating LPCs, diet may actually play a more important role.

In order to provide relevance to the human condition, we examined plasma LPC levels in a small cohort of lean, obese and obese T2DM individuals. Consistent with our HFD rodent studies, we observed a general reduction in plasma LPCs from both the obese non-diabetic and T2DM individuals. Although contrasting the reports of increased circulating LPC in obesity [6] and T2DM [23], these results are consistent with those of others who found that plasma LPCs were diminished in glucose intolerant and insulin resistant individuals [4], [7], [21]. Interestingly, we were unable to detect any differences in the LPC profile when comparing our obese otherwise healthy individuals with the T2DM patients. These findings may indicate that adiposity, rather than diabetes *per se*, makes the major contribution to the alterations in plasma LPC profile observed. Indeed, significant negative associations were found to exist between circulating LPCs and BMI. While we were unable to detect any difference in plasma insulin levels between groups we did observe a significant negative association between plasma LPCs and plasma insulin levels. Although our data lend support to the notion that obesity rather than diabetes may be the major factor leading to alterations in the plasma LPC profile, we cannot discount the influence of diet especially given the results obtained from our animal studies. Unfortunately, we do not have any information on the dietary habits of the individuals in our study so we cannot make any conclusions regarding the association between diet and plasma LPC levels. However, such a relationship needs to be examined in future studies. While our study shows that LPCs are reduced in the circulation of obese and obese T2DM individuals, it is important to note that our analysis was performed on a very small number of individuals. This relatively small sample size may have limited our ability to detect differences between groups. Therefore, further studies using large cohorts are required to identify if any differences in the plasma LPC profile exist between obese and obese T2DM individuals and to further explore the role of diet in influencing LPC levels.

Recently it has been demonstrated that LPC can exert beneficial effects on glucose metabolism [12]. Treating cultured adipocytes with LPC stimulated glucose uptake in a dose-dependent manner via an insulin-independent mechanism involving activation of protein kinase C δ [12]. Furthermore, these authors were also able to show that acute LPC treatment improved glycemia in mouse models of diabetes [12]. Thus, LPC may be a novel insulin independent signal that regulates blood glucose levels [12] and the reduction in circulating LPC observed in our obese mice and humans may play some role in contributing to the defects in glucose homeostasis observed in obesity and T2DM. This notion is particularly important since the concept of lipids exerting beneficial metabolic effects has recently received prominence [24].

In conclusion, our data provide evidence that marked changes occur in the plasma lipidome in the high fat fed mouse model of obesity. The most striking observation was the generalized reduction in plasma LPC species in fat fed mice. Our time course studies revealed that these alterations occur rapidly in response to fat feeding and coincide with increases in fat mass and the development of glucose intolerance and hyperinsulinemia. While our data show that adiposity was associated with LPC levels, diet appeared to be the most important determinant in these rodent studies. Consistent with our rodent model, our human data revealed a widespread reduction in LPC species in plasma from both obese and obese T2DM individuals. Interestingly, we were unable to detect any differences when comparing the obese otherwise healthy individuals with the obese T2DM patients. Therefore, our data indicate that diet and adiposity, rather than insulin resistance and diabetes *per se*, play an important role in altering the plasma LPC profile.

## Materials and Methods

### Ethics Statement

All animal experiments were approved by the Alfred Medical Research and Education Precinct Animal Ethics Committee and were in accordance with the National Health and Medical Research Council of Australia Guidelines on Animal Experimentation. The human study was approved by The Alfred Human Research Ethics Committee and each participant provided written informed consent.

### Changes in Plasma Lipids with Chronic High-fat Feeding

Eight week old male C57Bl/6J mice were obtained in-house from AMREP Animal Services and were fed either a low fat diet (LFD; N = 6; 14.3 MJ/kg, 76% of kJ from carbohydrate, 4.5% fat, 19% protein, Specialty Feeds, Glen Forrest, WA, Australia) or high-fat diet (HFD; N = 8; 19 MJ/kg, 35% of kJ from carbohydrate, 42% fat, 23% protein, Specialty Feeds, Glen Forrest, WA, Australia) for 12 weeks. The fatty acid profile of the diets is presented in Table S7. Animals were housed in a temperature controlled room (22±1°C) on a 12 hour light/dark cycle with free access to food and water. Four days prior to completion of the study, body composition (lean and fat mass) was determined using the EchoMRI 4-in-1 (Echo Medical Systems, Houston, TX, USA). At the end of the 12 week diet, mice were fasted for 5 hours and a blood sample (**∼**50 µL) was obtained, placed in EDTA-tubes and immediately put on ice. Samples were spun in a centrifuge (1500 g, 10 min, 4°C) and plasma was stored at −80°C for subsequent lipidomic and metabolite analysis. Mice were then anesthetized and tissues (liver, epididymal fat and muscle) were collected, immediately frozen in liquid nitrogen and stored at −80°C. HOMA-IR was calculated using the following formula: fasting glucose (mmol/L) × fasting insulin (µU/ml)/22.5.

### Time Course Changes in Plasma Lipids with High-fat Feeding

Eight week old male C57Bl/6J mice obtained from AMREP Animal Services were maintained on a HFD (N = 11) for 6 weeks. Lean and fat mass was determined prior to the commencement of the HFD (0 week) and again at 1, 3 and 6 weeks of HFD. Intraperitoneal glucose tolerance tests (1 g/kg lean body mass) were performed on 5 hour fasted mice at 0, 1, 3 and 6 weeks of HFD. Blood samples were obtained from the tail tip at the indicated times and glucose was measured using a glucometer (AccuCheck II; Roche, NSW, Australia). For subsequent analysis, the blood sample (**∼**50 µL) obtained prior to the glucose tolerance test was placed in EDTA-tubes and immediately stored on ice. Samples were spun in a centrifuge (1500 g, 10 min, 4°C) and plasma was stored at −80°C.

### Plasma Lipidomic Changes in Human Obesity and T2DM

Plasma samples from 30 male subjects were obtained from the Baker IDI Heart and Diabetes Institute Biobank. Subjects were classified as lean (N = 11; BMI<25), obese non-diabetic (N = 10; BMI>27) or obese T2DM (N = 9; BMI>27). The obese non-diabetic and obese T2DM groups were matched for body mass and BMI. Subjects in the obese T2DM group were being treated with diet (N = 6) or oral hypoglycaemic agents (N = 1 sulfonylurea; N = 2 metformin). In addition, 3 subjects in the T2DM group were being treated with anti-hypertensive medication. The T2DM individuals had no other medical conditions. Apart from those mentioned above, all were non-medicated and had no known existing medical conditions. Samples were collected after an overnight (10–12 hour) fast. Samples were collected from the antecubital vein into EDTA-vacutainer tubes (Becton Dickinson, North Ryde, NSW, Australia) and placed on ice. Samples were spun in a centrifuge (1500 g, 10 min, 4°C) and plasma was aliquoted and stored at −80°C for subsequent analysis.

### Lipidomic Analysis

Lipid analysis was performed by liquid chromatography, electrospray ionisation-tandem mass spectrometry (LC ESI-MS/MS) as described previously [26]–[29]. For tissue lipids, samples were homogenized in 300 ul 1xPBS, pH 7.47 and protein content was determined. Lipids were extracted from 25 µg of protein for liver, 10 µg of protein for adipose tissue and 50 µg of protein for muscle. For plasma analysis, lipids were extracted from 10 µl of plasma. Lipids were extracted using a single-phase chloroform: methanol (2∶1; 20 volumes) extraction following the addition of internal standards (100 pmol each of ceramide (Cer) 17∶0, glucosylceramide (monohexosylceramide, MHC) 16∶0 *d3,* lactosylceramide (dihexosylceramide, DHC) 16∶0 *d3* and trihexosylceramide (THC) 17∶0 (Matreya Inc., Pleasant Gap, USA), phosphatidylcholine (PC) 13∶0/13∶0, lysophosphatidylcholine (LPC) 13∶0, phosphatidylethanolamine (PE) 17∶0/17∶0, phosphatidylglycerol (PG) 17∶0/17∶0, bismonoacylglycerolphosphate (BMP) 14∶0/14∶0, sphingomyelin (SM) 12∶0 and sphingosine (Sph) 17∶1 (Avanti Polar Lipids, Alabaster, USA), together with 200 pmol of diacylglycerol (DG) 15∶0/15∶0 and 100 pmol of triacylglycerol (TG) 17∶0/17∶0/17∶0 (Sigma Aldrich). Samples were vortexed, mixed for 10 min on a rotation mixer, sonicated for 30 min and left to stand at room temperature for a further 20 min. Extracts were then centrifuged at 13,000 xg for 10 min, the supernatant was transferred to a clean tube and dried under nitrogen at 40°C. Lipids were redissolved in 50 µL water saturated butanol containing 10 mM NH_4_COOH then 50 µL methanol containing 10 mM NH_4_COOH was added.

Analysis was performed by electrospray ionisation-tandem mass spectrometry using a PE Sciex API 4000 Q/TRAP mass spectrometer with a turbo-ionspray source and Analyst 1.5 data system. Prior liquid chromatographic separation was performed on a Zorbax C18, 1.8 um, 50×2.1 mm column at 300 µL/minute using the following gradient conditions; 0% B to 100% B over 8.0 minutes followed by 2.5 minutes at 100% B, a return to 0% B over 0.5 minute then 3.0 minutes at 0% B prior to the next injection. DGs and TGs were separated using the same system with an isocratic flow of 15% A/85% B. Solvent A and B consisted of tetrahydrofuran:methanol:water in the ratios (30∶20∶50) and (75∶20∶5) respectively, both containing 10 mM NH_4_COOH.

Quantification of individual lipid species was performed using scheduled multiple-reaction monitoring (MRM) in positive ion mode. Individual lipid species monitored were the major species (greater than 1% of total) identified in human plasma. MRM experiments were based on product ion of *m/z* 264 [sphingosine–H_2_O]^+^ for Cer, MHC, DHC and THC, *m/z* 184 [phosphocholine]^+^ for SM, PC and LPC, *m/z* 189 [glycerophosphate-NH_4_]^+^ for PG and neutral loss (NL) of 141 Da for PE. DGs and TGs were monitored by the neutral loss of individual fatty acid species and BMP by the neutral loss corresponding to the monoacylglycerophosphate moiety. Each ion pair was monitored for 10–50 ms with a resolution of 0.7 amu at half-peak height and averaged from continuous scans over the elution period. Lipid concentrations were calculated by relating the peak area of each species to the peak area of the corresponding internal standard. Total lipids of each class were calculated by summing the individual lipid species.

### Blood Biochemistry

Plasma insulin was determined by an enzyme-linked immunosorbent assay (Millipore, St. Charles, MO, USA). Plasma FFAs were measured using an enzymatic colorimetric method (NEFA C test kit, Wako, Richmond, VA, USA). Plasma TGs, total cholesterol, LDL and HDL cholesterol were determined using a Cholestech LDX (Cholestech, Hayward, CA, USA).

### Statistical Analysis

For the 12 week HFD study, data were analysed using a Student’s *t*-test. Characteristic data are presented as the mean ± SEM. Lipidomic data are presented as the mean fold change from the appropriate control group. A value of p<0.05 was considered statistically significant. For the time course study, data were analysed using factorial repeated measures ANOVA. The associations between lipid (LPC species) and diet in mice were assessed using linear regression with diet (low fat or high fat) included as a categorical predictor. The influence of HOMA-IR on the associations was tested by including both diet and HOMA-IR in the model. For the human study, the associations between plasma lipids and disease status (lean, obese non-diabetic or obese T2DM) were assessed using linear regression with disease status fitted as a categorical predictor. Stepwise regression was also used to determine whether adjustment for age, total cholesterol, blood glucose, plasma FFA, insulin, weight or BMI influenced the associations. A significance value of p<0.05 was used to retain adjustment variables in the model. Spearman correlation coefficients were calculated between LPC levels and indices of metabolic homeostasis.

## Supporting Information

Table S1 Plasma lipid species measured in 12 week low and high fat-fed mice.
(DOC)

Table S2 Time course changes in the plasma lipidome in fat-fed mice.
(DOC)

Table S3 Relationship between plasma LPC levels and HOMA-IR in high fat fed mice.
(DOC)

Table S4 Relationship between plasma LPC levels and body composition in high fat fed mice.
(DOC)

Table S5 Relationship between plasma LPC levels, body composition and diet in high fat fed mice.
(DOC)

Table S6 Correlations between BMI and circulating insulin levels with plasma LPC species in the human cohort.
(DOC)

Table S7 The fatty acid profile of the low and high fat diet.
(DOC)
