# Supplementary material for: Plasma Lysophosphatidylcholine Levels Are Reduced in Obesity and Type 2 Diabetes
Source: PLoS One. 2012 Jul 25;7(7):e41456. doi: 10.1371/journal.pone.0041456 (PMC3405068; doi:10.1371/journal.pone.0041456)
Supplement: Table S1 — Plasma lipid species measured in 12 week low and high fat-fed mice. (DOC) [file pone.0041456.s001.doc]

**Table S1. Plasma lipid species measured in mice fed a low or high fat diet for 12 weeks.**

| **Lipid species (pmol/ml)** | **Low fat diet** | | **High fat diet** | | **p value** |
| --- | --- | --- | --- | --- | --- |
|  | **Mean** | **SEM** | **Mean** | **SEM** |  |
| **TG 28:0** | 2301.9 | 409.9 | 5188.9 | 984.4 | 0.034 |
| **TG 32:0** | 107.5 | 31.5 | 294.3 | 55.7 | 0.021 |
| **TG 36:0** | 171.1 | 29.6 | 377.0 | 71.0 | 0.039 |
| **TG 42:0** | 441.6 | 96.9 | 969.8 | 189.5 | 0.065 |
| **TG 44:0** | 707.5 | 215.2 | 2061.4 | 531.2 | 0.082 |
| **TG 46:0** | 132.6 | 31.6 | 639.7 | 190.1 | 0.055 |
| **TG 48:3** | 973.6 | 303.2 | 2082.7 | 841.3 | 0.401 |
| **TG 48:2** | 2090.0 | 878.6 | 4588.2 | 1550.2 | 0.279 |
| **TG 48:1** | 13201.1 | 7153.2 | 16542.5 | 4090.1 | 0.747 |
| **TG 48:0** | 392.6 | 124.5 | 1372.0 | 323.1 | 0.034 |
| **TG 50:4** | 1031.3 | 205.4 | 2703.1 | 1020.8 | 0.270 |
| **TG 50:3** | 6730.0 | 1900.6 | 11350.2 | 3219.9 | 0.302 |
| **TG 50:2** | 22450.4 | 6040.9 | 35966.5 | 7500.6 | 0.216 |
| **TG 50:1** | 14007.2 | 4419.7 | 41665.5 | 11205.9 | 0.086 |
| **TG 50:0** | 1598.9 | 363.9 | 4571.1 | 837.3 | 0.007 |
| **TG 52:4** | 21384.6 | 3958.8 | 37351.3 | 5485.7 | 0.145 |
| **TG 52:3** | 39892.6 | 6439.9 | 87884.4 | 14401.9 | 0.041 |
| **TG 52:2** | 64731.8 | 11251.6 | 125552.3 | 18953.5 | 0.027 |
| **TG 52:1** | 13643.8 | 2902.2 | 60987.3 | 14337.1 | 0.019 |
| **TG 54:6** | 3437.8 | 583.2 | 8289.7 | 2547.5 | 0.193 |
| **TG 54:5** | 7670.5 | 1294.6 | 18162.1 | 4705.1 | 0.107 |
| **TG 54:4** | 4204.2 | 744.1 | 12430.4 | 3503.3 | 0.086 |
| **TG 54:3** | 58566.5 | 12561.6 | 117139.7 | 24861.2 | 0.048 |
| **TG 54:2** | 12276.4 | 2992.5 | 45198.6 | 12107.9 | 0.032 |
| **TG 54:1** | 1935.5 | 475.2 | 10850.2 | 2985.1 | 0.025 |
| **TG 54:0** | 532.6 | 98.5 | 2361.9 | 557.3 | 0.010 |
| **TG 56:8** | 809.9 | 169.1 | 2025.8 | 343.9 | 0.043 |
| **TG 56:6** | 4556.9 | 882.5 | 14369.7 | 2491.9 | 0.006 |
| **DG 14:0 14:0** | 79.8 | 11.9 | 88.1 | 10.4 | 0.843 |
| **DG 14:1 16:0** | 75.4 | 12.8 | 86.3 | 9.5 | 0.758 |
| **DG 14:0 16:0** | 257.4 | 41.0 | 295.3 | 28.7 | 0.707 |
| **DG 14:0 18:2** | 65.7 | 7.4 | 92.0 | 12.0 | 0.161 |
| **DG 14:0 18:1** | 137.2 | 17.3 | 192.3 | 24.3 | 0.126 |
| **DG 16:0 16:0** | 1373.6 | 173.5 | 1729.7 | 100.6 | 0.237 |
| **DG 16:1 18:1** | 667.4 | 103.0 | 530.0 | 48.6 | 0.493 |
| **DG 16:0 18:2** | 714.7 | 82.4 | 1107.1 | 158.1 | 0.060 |
| **DG 16:0 18:1** | 1039.6 | 132.4 | 1674.5 | 224.4 | 0.024 |
| **DG 18:0 16:1** | 72.1 | 7.2 | 91.4 | 8.8 | 0.107 |
| **DG 16:0 18:0** | 890.1 | 97.6 | 1255.0 | 84.9 | 0.082 |
| **DG 18:2 18:2** | 660.1 | 72.1 | 698.4 | 102.5 | 0.891 |
| **DG 18:1 18:3** | 202.9 | 22.2 | 206.4 | 25.2 | 0.961 |
| **DG 16:0 20:4** | 60.3 | 9.8 | 189.7 | 46.2 | 0.031 |
| **DG 16:0 20:3** | 195.9 | 53.1 | 341.2 | 76.2 | 0.218 |
| **DG 18:1 18:2** | 1415.2 | 169.0 | 1537.8 | 174.2 | 0.467 |
| **DG 18:1 18:1** | 3511.9 | 524.2 | 3985.9 | 305.6 | 0.173 |
| **DG 18:0 18:2** | 187.4 | 19.7 | 343.8 | 48.6 | 0.016 |
| **DG 16:0 20:0** | 37.9 | 5.7 | 47.1 | 4.5 | 0.293 |
| **DG 18:0 18:0** | 1158.8 | 102.0 | 1346.8 | 30.0 | 0.252 |
| **DG 16:0 22:6** | 69.6 | 7.7 | 114.4 | 13.8 | 0.013 |
| **DG 16:0 22:5** | 37.2 | 3.2 | 53.9 | 4.4 | 0.032 |
| **DG 18:1 20:4** | 292.6 | 28.0 | 694.3 | 94.6 | 0.002 |
| **DG 18:1 20:3** | 117.8 | 23.0 | 190.8 | 13.0 | 0.006 |
| **DG 18:0 20:4** | 122.9 | 37.3 | 138.8 | 21.9 | 0.422 |
| **DG 18:1 20:0** | 33.7 | 6.2 | 44.0 | 3.7 | 0.125 |
| **Cer 16:0** | 167.7 | 32.6 | 260.7 | 39.9 | 0.112 |
| **Cer 18:0** | 36.6 | 6.7 | 69.7 | 11.9 | 0.048 |
| **Cer 20:0** | 46.3 | 5.2 | 141.0 | 25.2 | 0.008 |
| **Cer 22:0** | 360.5 | 60.9 | 704.4 | 108.7 | 0.027 |
| **Cer 24:1** | 551.7 | 47.9 | 524.9 | 56.1 | 0.734 |
| **Cer 24:0** | 464.0 | 54.3 | 440.4 | 41.7 | 0.731 |
| **Sph 18:1** | 176.8 | 28.3 | 175.2 | 34.6 | 0.758 |
| **MHC 16:0** | 467.5 | 47.6 | 853.3 | 87.4 | 0.001 |
| **MHC 18:1** | 26.2 | 3.1 | 30.3 | 2.8 | 0.295 |
| **MHC 18:0** | 105.1 | 18.8 | 276.1 | 37.3 | 0.001 |
| **MHC 20:0** | 601.6 | 41.4 | 2624.3 | 504.1 | 0.001 |
| **MHC 22:0** | 4407.4 | 525.2 | 11413.8 | 1890.9 | 0.002 |
| **MHC 24:1** | 4417.5 | 145.2 | 4871.6 | 599.1 | 0.167 |
| **MHC 24:0** | 2114.5 | 113.0 | 2841.9 | 332.9 | 0.029 |
| **DHC 16:0** | 84.6 | 8.3 | 192.1 | 21.1 | 0.002 |
| **DHC 18:1** | 18.9 | 1.4 | 20.9 | 1.0 | 0.131 |
| **DHC 18:0** | 3.2 | 1.1 | 5.1 | 1.8 | 0.517 |
| **DHC 20:0** | 2.7 | 1.1 | 1.6 | 0.4 | 0.356 |
| **DHC 22:0** | 24.7 | 2.6 | 45.6 | 2.7 | 0.001 |
| **THC 22:0** | 3.6 | 1.4 | 3.7 | 0.4 | 0.870 |
| **THC 24:1** | 7.8 | 0.9 | 8.8 | 1.9 | 0.925 |
| **THC 24:0** | 6.1 | 1.5 | 7.8 | 0.8 | 0.370 |
| **SM 14:0** | 63270.1 | 5088.7 | 155276.8 | 11547.4 | 0.000 |
| **SM 15:0** | 266508.0 | 22312.8 | 370634.6 | 26646.8 | 0.016 |
| **SM 16:1** | 726936.3 | 33769.7 | 785557.7 | 64280.8 | 0.569 |
| **SM 18:1** | 160117.9 | 17823.3 | 315207.2 | 45673.5 | 0.013 |
| **SM 18:0** | 319080.4 | 36747.6 | 622597.4 | 72070.4 | 0.007 |
| **SM 20:1** | 1065337.4 | 149225.1 | 903977.6 | 59349.5 | 0.475 |
| **SM 22:1** | 2394401.2 | 269086.6 | 2237824.3 | 148991.0 | 0.650 |
| **SM 22:0** | 2757687.0 | 154541.2 | 2494190.9 | 148063.8 | 0.160 |
| **SM 24:2** | 2277986.3 | 105658.0 | 2485693.0 | 84815.6 | 0.365 |
| **SM 24:0** | 622066.8 | 53248.6 | 517263.6 | 59312.5 | 0.091 |
| **SM 25:1** | 41372.2 | 2502.4 | 39270.3 | 4918.3 | 0.995 |
| **SM 26:1** | 41372.2 | 2502.4 | 39270.3 | 4918.3 | 0.995 |
| **SM 12:0** | 296530.3 | 13234.5 | 261220.7 | 12875.0 | 0.033 |
| **SM 16:0** | 1727866.0 | 83663.0 | 1765857.2 | 53691.9 | 0.862 |
| **SM 20:0** | 1850303.0 | 92573.0 | 1783969.5 | 130706.4 | 0.376 |
| **SM 24:1** | 2269794.7 | 126944.5 | 1578459.0 | 63046.7 | 0.000 |
| **PC 30:2** | 462.4 | 34.2 | 556.8 | 39.7 | 0.110 |
| **PC 30:1** | 1686.9 | 106.0 | 2069.1 | 51.8 | 0.004 |
| **PC 30:0** | 234.4 | 22.1 | 275.7 | 18.0 | 0.169 |
| **PC 32:2** | 617.0 | 26.9 | 655.8 | 30.2 | 0.375 |
| **PC 32:1** | 2333.9 | 57.1 | 2293.9 | 47.3 | 0.597 |
| **PC 32:0** | 1990.5 | 123.7 | 2158.5 | 81.5 | 0.260 |
| **PC 34:3** | 2979.7 | 285.8 | 3329.4 | 308.4 | 0.437 |
| **PC 34:2** | 3169.4 | 73.2 | 3432.0 | 119.5 | 0.111 |
| **PC 34:1** | 2337.0 | 32.9 | 2242.4 | 105.0 | 0.467 |
| **PC 34:0** | 2095.5 | 12.2 | 2083.8 | 18.8 | 0.640 |
| **PC 36:5** | 2951.6 | 41.7 | 3047.2 | 234.5 | 0.735 |
| **PC 36:4** | 3543.5 | 133.8 | 4024.4 | 160.1 | 0.048 |
| **PC 36:3** | 2147.1 | 98.1 | 2762.9 | 139.2 | 0.006 |
| **PC 36:2** | 2139.0 | 90.5 | 1970.7 | 62.4 | 0.139 |
| **PC 36:1** | 2576.7 | 53.0 | 2662.7 | 178.2 | 0.693 |
| **PC 38:6** | 1499.4 | 33.5 | 2786.1 | 518.5 | 0.055 |
| **PC 38:5** | 4463.9 | 135.3 | 2021.9 | 509.1 | 0.002 |
| **PC 38:4** | 1693.1 | 268.2 | 1599.0 | 105.4 | 0.724 |
| **PC 38:3** | 2110.1 | 167.1 | 2076.0 | 247.5 | 0.918 |
| **PC 38:2** | 988.6 | 444.6 | 807.9 | 79.3 | 0.652 |
| **PC 40:7** | 2202.6 | 131.6 | 2406.9 | 68.8 | 0.165 |
| **PC 40:6** | 3019.0 | 119.9 | 3415.4 | 84.7 | 0.016 |
| **PC 40:5** | 1905.9 | 68.7 | 1936.8 | 165.2 | 0.881 |
| **PC 44:12** | 18.2 | 1.3 | 20.4 | 1.6 | 0.354 |
| **BMP 16:0 16:1** | 14.2 | 3.4 | 3.3 | 0.4 | 0.003 |
| **BMP 16:0 16:0** | 333.0 | 68.0 | 272.8 | 20.3 | 0.380 |
| **BMP 16:1 18:1** | 12.3 | 2.3 | 10.7 | 2.1 | 0.728 |
| **BMP 16:0 18:1** | 6.0 | 1.4 | 6.4 | 1.6 | 0.683 |
| **BMP 16:0 18:0** | 2.7 | 0.7 | 3.6 | 1.0 | 0.694 |
| **BMP 18:1 18:2** | 18.7 | 2.4 | 31.2 | 2.5 | 0.002 |
| **BMP 18:1 18:1** | 22.1 | 1.8 | 33.2 | 5.0 | 0.032 |
| **BMP 18:0 18:1** | 3.9 | 1.0 | 10.2 | 2.5 | 0.065 |
| **BMP 16:0 22:6** | 2.9 | 0.7 | 4.4 | 0.8 | 0.224 |
| **BMP 18:2 22:6** | 25.9 | 1.5 | 22.0 | 2.4 | 0.771 |
| **BMP 18:1 22:6** | 24.5 | 2.7 | 28.6 | 2.8 | 0.240 |
| **BMP 18:1 22:5** | 3.6 | 0.9 | 5.8 | 1.1 | 0.112 |
| **BMP 20:4 22:6** | 16.6 | 2.2 | 19.0 | 2.2 | 0.278 |
| **BMP 22:6 22:6** | 30.9 | 2.6 | 67.5 | 6.5 | 0.002 |
| **BMP 22:5 22:6** | 5.4 | 1.9 | 12.3 | 1.6 | 0.010 |
| **BMP 22:5 22:5** | 1.8 | 0.3 | 4.0 | 0.7 | 0.056 |
| **PG 16:1 18:1** | 71.4 | 11.8 | 176.4 | 17.1 | 0.001 |
| **PG 16:0 18:1** | 102.9 | 19.8 | 126.2 | 17.0 | 0.138 |
| **PG 18:1 18:1** | 81.8 | 15.1 | 261.7 | 26.3 | 0.000 |
| **PG 18:0 18:1** | 88.0 | 15.9 | 130.9 | 20.5 | 0.038 |
| **PE 32:2** | 9.0 | 1.7 | 4.0 | 0.7 | 0.030 |
| **PE 32:1** | 14.2 | 5.6 | 8.2 | 2.2 | 0.377 |
| **PE 32:0** | 17.3 | 2.9 | 14.9 | 2.2 | 0.947 |
| **PE 34:2** | 531.2 | 87.0 | 394.5 | 41.7 | 0.130 |
| **PE 34:1** | 222.6 | 29.8 | 165.3 | 16.2 | 0.171 |
| **PE 34:0** | 1385.8 | 10.0 | 1360.9 | 17.7 | 0.213 |
| **PE 36:5** | 155.4 | 26.6 | 88.0 | 11.3 | 0.147 |
| **PE 36:4** | 756.8 | 147.1 | 748.8 | 47.9 | 0.900 |
| **PE 36:3** | 510.3 | 61.7 | 461.0 | 62.2 | 0.803 |
| **PE 36:2** | 1412.4 | 235.5 | 2094.3 | 294.7 | 0.100 |
| **PE 36:1** | 447.4 | 64.6 | 659.2 | 82.5 | 0.042 |
| **PE 36:0** | 49.0 | 9.4 | 59.5 | 15.6 | 0.573 |
| **PE 38:6** | 1527.8 | 196.2 | 1887.6 | 338.2 | 0.399 |
| **PE 38:5** | 961.4 | 94.9 | 960.9 | 36.2 | 0.494 |
| **PE 38:4** | 1840.7 | 228.1 | 3351.6 | 191.9 | 0.000 |
| **PE 38:3** | 418.5 | 53.3 | 581.1 | 30.1 | 0.015 |
| **PE 38:2** | 115.2 | 18.7 | 107.1 | 7.7 | 0.911 |
| **PE 38:1** | 48.2 | 7.1 | 62.0 | 6.5 | 0.102 |
| **PE 38:0** | 149.1 | 9.4 | 205.5 | 12.3 | 0.004 |
| **PE 40:7** | 339.8 | 30.3 | 327.0 | 51.4 | 0.827 |
| **PE 40:6** | 543.6 | 52.6 | 857.6 | 167.6 | 0.148 |
| **LPC 14:0** | 427.0 | 25.7 | 284.6 | 21.1 | 0.003 |
| **LPC 15:0** | 443.6 | 19.7 | 290.0 | 11.5 | 0.000 |
| **LPC 16:1** | 4644.4 | 432.1 | 2120.0 | 63.6 | 0.001 |
| **LPC 16:0** | 35774.1 | 1254.8 | 30753.3 | 849.5 | 0.011 |
| **LPC 18:2** | 33983.7 | 2234.7 | 29283.5 | 1256.3 | 0.193 |
| **LPC 18:1** | 25480.1 | 1322.8 | 19478.8 | 504.2 | 0.038 |
| **LPC 18:0** | 17502.8 | 391.7 | 18689.6 | 377.9 | 0.039 |
| **LPC 20:5** | 1488.6 | 271.0 | 660.8 | 69.0 | 0.018 |
| **LPC 20:4** | 19984.1 | 647.4 | 22686.2 | 475.3 | 0.006 |
| **LPC 20:3** | 5979.1 | 699.3 | 5036.4 | 708.1 | 0.744 |
| **LPC 20:2** | 633.8 | 75.1 | 627.3 | 82.8 | 0.478 |
| **LPC 20:1** | 1313.7 | 57.8 | 698.4 | 72.5 | 0.009 |
| **LPC 20:0** | 802.8 | 91.1 | 598.0 | 55.0 | 0.062 |
| **LPC 22:6** | 13355.5 | 691.4 | 13774.9 | 660.1 | 0.326 |
