# Supplementary material for: Plasma Lysophosphatidylcholine Levels Are Reduced in Obesity and Type 2 Diabetes
Source: PLoS One. 2012 Jul 25;7(7):e41456. doi: 10.1371/journal.pone.0041456 (PMC3405068; doi:10.1371/journal.pone.0041456)
Supplement: Table S2 — Time course changes in the plasma lipidome in fat-fed mice. (DOC) [file pone.0041456.s002.doc]

**Table S2.** Time course changes in the plasma lipidome in fat-fed mice.

| **Lipid species** | **0** | | **1** | | **3** | | **6** | | **Main effect for time** | **0 v 1** | **0 v 3** | **0 v 6** | **1 v 3** | **1 v 6** | **3 v 6** |
| --- | --- | --- | --- | --- | --- | --- | --- | --- | --- | --- | --- | --- | --- | --- | --- |
| **(pmol/ml)** | **Mean** | **SEM** | **Mean** | **SEM** | **Mean** | **SEM** | **Mean** | **SEM** | **p value** | **p value** | **p value** | **p value** | **p value** | **p value** | **p value** |
| **TG 14:0 16:0 18:2** | 1398.5 | 208.0 | 602.5 | 79.6 | 900.5 | 137.9 | 608.3 | 46.3 | <0.001 | 0.001 | 0.014 | <0.001 | 0.280 | 0.976 | 0.138 |
| **TG 14:0 16:1 18:1** | 659.0 | 75.5 | 368.9 | 47.4 | 527.5 | 67.5 | 388.7 | 28.6 | 0.006 | 0.010 | 0.132 | 0.009 | 0.166 | 0.817 | 0.113 |
| **TG 14:0 16:1 18:2** | 203.8 | 18.5 | 110.4 | 12.1 | 144.8 | 20.4 | 117.1 | 10.9 | 0.002 | 0.003 | 0.019 | 0.003 | 0.332 | 0.780 | 0.254 |
| **TG 14:0 18:0 18:1** | 46.2 | 4.0 | 92.0 | 13.5 | 104.6 | 16.0 | 66.9 | 8.0 | 0.006 | 0.024 | 0.007 | 0.217 | 0.451 | 0.135 | 0.071 |
| **TG 14:0 18:2 18:2** | 149.5 | 11.3 | 97.3 | 8.3 | 124.3 | 22.5 | 103.9 | 15.7 | 0.104 | * | * | * | * | * | * |
| **TG 14:1 16:0 18:1** | 253.0 | 21.6 | 150.9 | 15.3 | 184.7 | 22.4 | 136.9 | 9.6 | <0.001 | 0.001 | 0.014 | <0.001 | 0.203 | 0.593 | 0.174 |
| **TG 14:1 16:1 18:0** | 2716.1 | 430.9 | 991.6 | 137.5 | 1413.2 | 217.1 | 1086.3 | 71.9 | <0.001 | <0.001 | <0.001 | <0.001 | 0.458 | 0.788 | 0.357 |
| **TG 14:1 18:0 18:2** | 72.2 | 4.6 | 48.4 | 4.1 | 55.2 | 4.6 | 45.0 | 4.0 | 0.001 | 0.003 | 0.014 | 0.001 | 0.300 | 0.605 | 0.271 |
| **TG 14:1 18:1 18:1** | 1025.4 | 120.5 | 450.3 | 54.1 | 682.1 | 84.0 | 498.0 | 45.1 | <0.001 | <0.001 | 0.0 | <0.001 | 0.148 | 0.694 | 0.136 |
| **TG 15:0 18:1 16:0** | 250.3 | 18.3 | 144.4 | 13.2 | 163.7 | 21.1 | 130.9 | 7.2 | <0.001 | <0.001 | <0.001 | <0.001 | 0.402 | 0.552 | 0.328 |
| **TG 15:0 18:1 18:1** | 257.7 | 11.4 | 133.1 | 8.0 | 150.4 | 15.8 | 124.5 | 13.3 | 0.531 | * | * | * | * | * | * |
| **TG 16:0 16:0 16:0** | 623.5 | 37.3 | 716.0 | 63.2 | 872.0 | 124.9 | 614.4 | 59.7 | 0.073 | * | * | * | * | * | * |
| **TG 16:0 16:0 18:0** | 505.5 | 34.9 | 1723.0 | 201.6 | 1666.3 | 300.4 | 1163.0 | 186.5 | <.001 | <0.001 | <0.001 | 0.026 | 0.842 | 0.132 | 0.084 |
| **TG 16:0 16:0 18:2** | 2844.9 | 151.9 | 2119.8 | 196.0 | 2556.8 | 429.8 | 1794.4 | 180.9 | 0.038 | 0.138 | 0.440 | 0.037 | 0.245 | 0.384 | 0.113 |
| **TG 16:0 18:0 18:1** | 1282.2 | 72.1 | 2763.0 | 297.4 | 3165.1 | 518.5 | 2022.1 | 235.3 | 0.001 | 0.008 | 0.002 | 0.114 | 0.384 | 0.114 | 0.045 |
| **TG 16:0 18:2 18:2** | 7181.0 | 501.9 | 4191.0 | 302.8 | 4560.4 | 888.0 | 4242.4 | 630.8 | 0.003 | 0.006 | 0.004 | 0.004 | 0.898 | 0.951 | 0.706 |
| **TG 16:1 16:1 16:1** | 291.2 | 48.0 | 112.7 | 14.6 | 146.8 | 19.4 | 125.5 | 8.2 | <0.001 | <0.001 | <0.001 | <0.001 | 0.633 | 0.732 | 0.571 |
| **TG 16:1 16:1 18:0** | 99.9 | 9.9 | 138.2 | 19.3 | 160.1 | 26.7 | 111.1 | 12.0 | 0.114 | * | * | * | * | * | * |
| **TG 16:1 18:1 18:1** | 5619.4 | 451.7 | 2161.2 | 294.7 | 3377.9 | 370.9 | 2687.6 | 219.1 | <0.001 | <0.001 | <0.001 | <0.001 | 0.033 | 0.261 | 0.143 |
| **TG 16:1 18:1 18:2** | 7435.6 | 487.9 | 3210.0 | 256.7 | 4292.8 | 527.5 | 3634.7 | 400.0 | <0.001 | <0.001 | <0.001 | <0.001 | 0.216 | 0.506 | 0.306 |
| **TG 17:0 16:0 16:1** | 654.3 | 49.4 | 395.2 | 34.4 | 447.4 | 57.3 | 373.5 | 21.1 | <0.001 | <0.001 | 0.002 | <0.001 | 0.393 | 0.721 | 0.447 |
| **TG 17:0 16:0 18:0** | 85.4 | 7.2 | 111.6 | 14.3 | 91.5 | 12.8 | 90.0 | 14.5 | 0.491 | * | * | * | * | * | * |
| **TG 17:0 18:1 14:0** | 406.2 | 35.0 | 231.4 | 21.1 | 247.7 | 34.0 | 208.5 | 13.5 | <0.001 | <0.001 | <0.001 | <0.001 | 0.680 | 0.564 | 0.583 |
| **TG 17:0 18:1 16:0** | 228.1 | 13.2 | 256.8 | 28.0 | 289.0 | 44.9 | 206.5 | 17.1 | 0.245 | * | * | * | * | * | * |
| **TG 17:0 18:1 16:1** | 1224.5 | 60.2 | 716.4 | 62.0 | 918.5 | 100.3 | 736.8 | 64.6 | <0.001 | <0.001 | 0.009 | <0.001 | 0.168 | 0.852 | 0.105 |
| **TG 17:0 18:1 18:1** | 702.2 | 32.3 | 493.6 | 31.8 | 587.0 | 59.3 | 496.0 | 38.9 | 0.006 | 0.011 | 0.074 | 0.007 | 0.304 | 0.969 | 0.154 |
| **TG 17:0 18:2 16:0** | 827.9 | 48.2 | 569.0 | 54.9 | 755.6 | 87.8 | 596.8 | 53.3 | 0.023 | 0.040 | 0.438 | 0.045 | 0.123 | 0.765 | 0.095 |
| **TG 18:0 18:0 18:0** | 26.3 | 2.9 | 221.7 | 67.5 | 137.5 | 15.6 | 111.2 | 27.9 | 0.006 | 0.003 | 0.086 | 0.102 | 0.089 | 0.105 | 0.607 |
| **TG 18:0 18:0 18:1** | 234.4 | 16.0 | 615.3 | 73.3 | 617.8 | 91.9 | 400.2 | 61.7 | <0.001 | <0.001 | 0.002 | 0.085 | 0.979 | 0.028 | 0.065 |
| **TG 18:0 18:1 18:1** | 1155.7 | 53.5 | 2354.9 | 213.6 | 2561.4 | 348.5 | 1851.0 | 223.3 | <0.001 | 0.003 | 0.001 | 0.044 | 0.536 | 0.138 | 0.097 |
| **TG 18:0 18:2 18:2** | 483.6 | 26.2 | 517.1 | 47.7 | 623.8 | 104.6 | 486.0 | 51.6 | 0.366 | * | * | * | * | * | * |
| **TG 18:1 14:0 16:0** | 643.4 | 55.5 | 532.7 | 72.2 | 706.9 | 107.1 | 474.9 | 38.1 | 0.136 | * | * | * | * | * | * |
| **TG 18:1 18:1 18:2** | 3824.0 | 186.5 | 2673.3 | 186.8 | 3159.1 | 447.4 | 2583.7 | 297.4 | 0.030 | 0.033 | 0.137 | 0.037 | 0.273 | 0.838 | 0.394 |
| **TG 18:1 18:1 20:4** | 1427.9 | 74.3 | 2122.9 | 132.8 | 2079.3 | 116.6 | 1616.4 | 92.4 | <0.001 | <0.001 | <0.001 | 0.170 | 0.747 | 0.002 | 0.002 |
| **TG 18:1 18:1 22:6** | 1568.5 | 89.7 | 1194.7 | 45.3 | 1167.6 | 148.9 | 1447.7 | 81.4 | 0.021 | 0.035 | 0.040 | 0.403 | 0.850 | 0.086 | 0.137 |
| **TG 18:1 18:2 18:2** | 3458.9 | 152.6 | 2475.7 | 189.8 | 2809.4 | 367.6 | 2321.5 | 256.7 | 0.011 | 0.019 | 0.065 | 0.011 | 0.333 | 0.653 | 0.335 |
| **TG 18:2 18:2 18:2** | 369.5 | 20.3 | 196.2 | 23.1 | 235.2 | 61.4 | 223.3 | 42.5 | 0.014 | 0.015 | 0.018 | 0.028 | 0.750 | 0.617 | 0.826 |
| **TG 18:2 18:2 20:4** | 330.2 | 21.6 | 641.2 | 48.3 | 521.1 | 57.7 | 415.4 | 43.4 | <0.001 | <0.001 | 0.002 | 0.096 | 0.022 | <0.001 | 0.041 |
| **DG 14:0 14:0** | 24.3 | 3.0 | 17.0 | 1.6 | 17.4 | 1.1 | 20.0 | 1.5 | 0.054 | * | * | * | * | * | * |
| **DG 14:0 16:0** | 182.2 | 36.6 | 197.3 | 29.0 | 209.4 | 25.5 | 213.0 | 19.0 | 0.937 | * | * | * | * | * | * |
| **DG 14:1 16:0** | 71.4 | 11.7 | 46.8 | 7.1 | 39.3 | 3.5 | 46.7 | 5.8 | 0.005 | 0.008 | 0.004 | 0.018 | 0.648 | 0.948 | 0.414 |
| **DG 16:0 16:0** | 617.0 | 239.0 | 1330.2 | 333.0 | 1437.7 | 333.9 | 1578.0 | 293.4 | 0.300 | * | * | * | * | * | * |
| **DG 14:0 18:1** | 126.7 | 16.5 | 95.1 | 9.5 | 92.3 | 8.5 | 88.7 | 7.8 | 0.007 | 0.016 | 0.011 | 0.008 | 0.813 | 0.609 | 0.733 |
| **DG 14:0 18:2** | 82.4 | 9.2 | 58.9 | 5.4 | 54.2 | 5.3 | 51.1 | 4.6 | <0.001 | 0.002 | 0.001 | <0.001 | 0.626 | 0.574 | 0.605 |
| **DG 16:0 18:0** | 580.4 | 307.5 | 1772.9 | 467.9 | 2215.0 | 610.7 | 2237.3 | 497.4 | 0.190 | * | * | * | * | * | * |
| **DG 16:0 18:1** | 1510.3 | 74.1 | 1346.8 | 100.7 | 1378.8 | 150.7 | 967.0 | 42.8 | <0.001 | 0.170 | 0.631 | <0.001 | 0.181 | <0.001 | 0.009 |
| **DG 18:0 16:1** | 61.4 | 6.0 | 44.9 | 4.5 | 47.9 | 5.8 | 41.6 | 2.5 | 0.004 | 0.014 | 0.020 | 0.003 | 0.588 | 0.415 | 0.367 |
| **DG 16:0 18:2** | 1272.0 | 53.9 | 1088.5 | 70.8 | 1008.9 | 128.8 | 829.4 | 67.0 | <0.001 | 0.015 | 0.031 | <0.001 | 0.927 | 0.093 | 0.047 |
| **DG 16:1 18:1** | 869.5 | 61.5 | 454.2 | 48.6 | 546.8 | 63.5 | 448.7 | 28.3 | <0.001 | <0.001 | <0.001 | <0.001 | 0.036 | 0.687 | 0.037 |
| **DG 16:0 20:0** | 31.5 | 6.3 | 48.5 | 8.7 | 48.1 | 7.1 | 51.4 | 7.9 | 0.540 | * | * | * | * | * | * |
| **DG 18:0 18:0** | 250.1 | 112.7 | 746.3 | 208.1 | 1121.7 | 355.9 | 1087.6 | 299.9 | 0.200 | * | * | * | * | * | * |
| **DG 18:0 18:1** | 153.8 | 15.5 | 254.2 | 20.9 | 237.9 | 32.5 | 176.5 | 27.4 | 0.002 | 0.006 | 0.008 | 0.429 | 0.750 | 0.025 | 0.020 |
| **DG 18:0 18:2** | 202.4 | 11.5 | 303.4 | 21.9 | 266.2 | 33.1 | 232.7 | 24.2 | 0.060 | * | * | * | * | * | * |
| **DG 18:1 18:1** | 1957.9 | 113.7 | 1403.8 | 99.0 | 1593.9 | 126.0 | 1170.2 | 62.4 | <0.001 | 0.003 | 0.307 | <0.001 | 0.013 | 0.128 | <0.001 |
| **DG 16:0 20:3** | 52.6 | 2.9 | 53.7 | 5.6 | 49.8 | 5.5 | 50.0 | 6.4 | <0.001 | * | * | * | * | * | * |
| **DG 18:1 18:2** | 2339.0 | 113.2 | 1694.3 | 120.8 | 1657.9 | 191.9 | 1204.2 | 67.2 | <0.001 | <0.001 | 0.002 | <0.001 | 0.403 | 0.006 | 0.002 |
| **DG 16:0 20:4** | 108.4 | 3.7 | 191.7 | 21.6 | 153.9 | 11.5 | 208.1 | 42.7 | 0.033 | 0.101 | 0.215 | 0.025 | 0.395 | 0.382 | 0.203 |
| **DG 18:1 18:3** | 292.5 | 19.9 | 236.5 | 19.1 | 223.9 | 28.5 | 146.4 | 8.5 | <0.001 | 0.016 | 0.008 | <0.001 | 0.895 | <0.001 | 0.001 |
| **DG 18:2 18:2** | 393.9 | 20.6 | 309.2 | 30.9 | 271.7 | 46.0 | 195.3 | 17.3 | <0.001 | 0.009 | 0.010 | <0.001 | 0.715 | 0.015 | 0.014 |
| **DG 18:0 20:4** | 181.5 | 9.1 | 256.9 | 19.8 | 162.4 | 9.3 | 173.7 | 25.4 | 0.006 | 0.006 | 0.849 | 0.885 | 0.008 | 0.012 | 0.692 |
| **DG 18:1 20:3** | 181.7 | 9.1 | 226.4 | 19.5 | 267.4 | 25.8 | 179.0 | 16.9 | <0.001 | 0.145 | <0.001 | 0.903 | 0.019 | 0.079 | <0.001 |
| **DG 16:0 22:5** | 60.0 | 6.8 | 77.8 | 7.1 | 106.2 | 16.7 | 86.1 | 11.4 | 0.049 | 0.420 | 0.035 | 0.415 | 0.116 | 0.645 | 0.121 |
| **DG 18:1 20:4** | 761.9 | 39.5 | 1137.2 | 76.1 | 1131.7 | 71.2 | 782.4 | 37.7 | <0.001 | <0.001 | <0.001 | 0.574 | 0.503 | <0.001 | <0.001 |
| **DG 16:0 22:6** | 136.0 | 7.4 | 141.1 | 14.3 | 143.5 | 11.5 | 167.0 | 31.2 | 0.422 | * | * | * | * | * | * |
| **Cer 16:0** | 135.8 | 7.7 | 185.2 | 14.4 | 223.6 | 12.8 | 206.2 | 23.5 | <0.001 | 0.001 | <0.001 | <0.001 | 0.023 | 0.134 | 0.212 |
| **Cer 18:0** | 37.3 | 2.4 | 53.2 | 4.1 | 57.1 | 2.9 | 60.9 | 7.9 | <0.001 | 0.007 | 0.003 | <0.001 | 0.481 | 0.339 | 0.479 |
| **Cer 20:0** | 63.8 | 3.5 | 121.4 | 11.2 | 122.9 | 10.1 | 136.4 | 21.1 | <0.001 | <0.001 | <0.001 | <0.001 | 0.914 | 0.556 | 0.358 |
| **Cer 22:0** | 929.7 | 80.6 | 1552.5 | 120.8 | 1458.3 | 80.6 | 1088.0 | 67.4 | <0.001 | <0.001 | <0.001 | 0.133 | 0.365 | <0.001 | 0.001 |
| **Cer 24:1** | 996.3 | 44.2 | 551.2 | 48.6 | 635.3 | 49.7 | 501.6 | 52.0 | <0.001 | <0.001 | <0.001 | <0.001 | 0.050 | 0.236 | 0.008 |
| **Cer 24:0** | 885.2 | 68.1 | 699.8 | 46.4 | 653.9 | 24.0 | 482.4 | 19.6 | <0.001 | 0.003 | 0.001 | <0.001 | 0.429 | 0.002 | 0.006 |
| **Sph 18:1** | 19.9 | 1.9 | 15.8 | 1.7 | 15.8 | 1.4 | 10.6 | 1.0 | <0.001 | 0.112 | 0.048 | <0.001 | 0.993 | 0.013 | 0.034 |
| **MHC 16:0** | 631.9 | 28.5 | 717.5 | 46.4 | 925.7 | 63.7 | 897.7 | 62.4 | <0.001 | 0.132 | <0.001 | <0.001 | 0.002 | 0.003 | 0.616 |
| **MHC 18:1** | 98.3 | 3.4 | 94.7 | 4.4 | 96.3 | 6.1 | 92.7 | 4.6 | 0.823 | * | * | * | * | * | * |
| **MHC 18:0** | 64.6 | 3.5 | 112.1 | 7.6 | 158.7 | 15.8 | 197.9 | 33.2 | <0.001 | 0.044 | <0.001 | <0.001 | 0.047 | 0.002 | 0.092 |
| **MHC 20:0** | 199.3 | 13.5 | 407.4 | 48.0 | 529.7 | 63.5 | 666.8 | 91.3 | <0.001 | 0.004 | <0.001 | <0.001 | 0.072 | 0.001 | 0.045 |
| **MHC 22:0** | 1781.9 | 73.9 | 3207.5 | 278.3 | 4164.9 | 314.3 | 3529.3 | 181.5 | <0.001 | <0.001 | <0.001 | <0.001 | 0.008 | 0.288 | 0.041 |
| **MHC 24:1** | 1789.0 | 69.6 | 1344.6 | 99.7 | 1749.8 | 156.9 | 1545.0 | 113.3 | 0.005 | 0.008 | 0.760 | 0.151 | 0.009 | 0.126 | 0.118 |
| **MHC 24:0** | 772.5 | 34.9 | 833.1 | 39.3 | 998.4 | 44.9 | 796.0 | 31.7 | <0.001 | 0.442 | <0.001 | 0.635 | 0.002 | 0.455 | <0.001 |
| **DHC 16:0** | 83.0 | 8.4 | 113.6 | 7.7 | 126.0 | 10.8 | 128.7 | 11.6 | 0.002 | 0.016 | 0.003 | 0.003 | 0.308 | 0.426 | 0.822 |
| **DHC 18:1** | 81.1 | 7.0 | 84.1 | 6.3 | 94.3 | 9.3 | 100.5 | 7.8 | 0.222 | * | * | * | * | * | * |
| **DHC 22:0** | 38.5 | 2.8 | 57.0 | 6.4 | 58.0 | 7.4 | 47.3 | 3.4 | 0.078 | * | * | * | * | * | * |
| **DHC 24:1** | 105.7 | 9.4 | 117.8 | 11.9 | 81.4 | 5.0 | 92.0 | 6.6 | 0.032 | 0.335 | 0.135 | 0.272 | 0.029 | 0.107 | 0.397 |
| **DHC 24:0** | 34.6 | 4.4 | 49.6 | 6.2 | 48.8 | 4.6 | 33.8 | 2.9 | 0.011 | 0.041 | 0.022 | 0.889 | 0.893 | 0.052 | 0.040 |
| **THC 16:0** | 39.8 | 3.9 | 44.2 | 4.7 | 47.0 | 4.7 | 39.9 | 3.9 | 0.463 | * | * | * | * | * | * |
| **THC 18:1** | 93.9 | 7.3 | 80.9 | 8.1 | 91.9 | 11.3 | 98.6 | 4.6 | 0.482 | * | * | * | * | * | * |
| **SM 14:0** | 515.5 | 40.6 | 1283.6 | 88.6 | 1329.4 | 83.6 | 1561.5 | 77.4 | <0.001 | <0.001 | <0.001 | <0.001 | 0.558 | 0.003 | 0.005 |
| **SM 15:0** | 2162.8 | 106.2 | 2911.8 | 211.1 | 3033.1 | 153.1 | 3643.0 | 191.1 | <0.001 | <0.001 | <0.001 | <0.001 | 0.527 | 0.002 | 0.003 |
| **SM 16:1** | 7806.5 | 272.5 | 9526.1 | 603.6 | 9013.4 | 308.4 | 9801.0 | 384.5 | 0.003 | 0.007 | 0.028 | 0.003 | 0.333 | 0.602 | 0.300 |
| **SM 16:0** | 35011.9 | 1958.0 | 45698.9 | 1945.9 | 44578.5 | 1836.7 | 49202.0 | 1120.2 | <0.001 | <0.001 | <0.001 | <0.001 | 0.687 | 0.139 | 0.129 |
| **SM 18:1** | 1686.1 | 89.6 | 2585.1 | 143.8 | 2618.6 | 122.2 | 3019.2 | 142.3 | <0.001 | <0.001 | <0.001 | <0.001 | 0.826 | 0.019 | 0.013 |
| **SM 18:0** | 3586.4 | 252.8 | 5677.5 | 335.9 | 5762.2 | 162.5 | 6475.0 | 315.4 | <0.001 | <0.001 | <0.001 | <0.001 | 0.813 | 0.079 | 0.054 |
| **SM 20:1** | 14455.9 | 502.1 | 14138.9 | 806.4 | 14831.4 | 521.1 | 13499.8 | 385.0 | 0.399 | * | * | * | * | * | * |
| **SM 22:1** | 76072.3 | 3623.9 | 71142.6 | 7997.3 | 79749.9 | 5783.0 | 75432.2 | 5653.7 | 0.534 | * | * | * | * | * | * |
| **SM 22:0** | 121604.9 | 4388.6 | 160374.6 | 8314.2 | 162303.9 | 6913.6 | 143524.3 | 3534.2 | <0.001 | <0.001 | <0.001 | 0.003 | 0.778 | 0.019 | 0.025 |
| **SM 24:1** | 61443.7 | 2516.7 | 54337.5 | 4010.2 | 54494.8 | 2574.4 | 57914.4 | 2869.6 | 0.127 | * | * | * | * | * | * |
| **SM 24:0** | 9043.0 | 368.8 | 8600.1 | 329.0 | 6594.8 | 341.9 | 6525.6 | 426.1 | <0.001 | 0.396 | <0.001 | <0.001 | <0.001 | 0.001 | 0.894 |
| **PC 30:2** | 1743.8 | 67.3 | 2202.3 | 127.7 | 2118.8 | 81.2 | 2208.3 | 80.8 | <0.001 | 0.001 | 0.003 | 0.002 | 0.475 | 0.960 | 0.721 |
| **PC 32:2** | 2404.4 | 125.0 | 2655.4 | 173.8 | 2961.2 | 90.3 | 2743.9 | 75.4 | 0.013 | 0.124 | 0.008 | 0.099 | 0.149 | 0.581 | 0.181 |
| **PC 32:1** | 112699.7 | 5101.4 | 82114.9 | 6800.9 | 95392.9 | 4717.8 | 80511.8 | 2838.0 | <0.001 | <0.001 | 0.005 | <0.001 | 0.025 | 0.777 | 0.033 |
| **PC 32:0** | 22551.1 | 983.5 | 24400.2 | 1044.7 | 23202.7 | 767.8 | 19932.5 | 625.4 | 0.003 | 0.235 | 0.562 | 0.025 | 0.290 | 0.002 | 0.017 |
| **PC 34:3** | 30337.6 | 1126.1 | 28597.5 | 1654.7 | 31144.5 | 803.0 | 26272.7 | 546.4 | 0.014 | 0.252 | 0.592 | 0.028 | 0.218 | 0.129 | 0.014 |
| **PC 34:2** | 5127.5 | 101.7 | 5085.0 | 204.3 | 4677.5 | 135.8 | 4121.0 | 145.2 | <0.001 | 0.842 | 0.100 | <0.001 | 0.063 | <0.001 | 0.013 |
| **PC 34:1** | 348502.2 | 10794.5 | 304030.3 | 11615.2 | 285884.3 | 7000.7 | 247885.9 | 9658.1 | <0.001 | 0.001 | <0.001 | <0.001 | 0.159 | <0.001 | 0.005 |
| **PC 36:5** | 17758.3 | 827.2 | 17568.6 | 1966.5 | 21958.2 | 1876.6 | 23914.6 | 1802.8 | <0.001 | 0.901 | 0.010 | 0.001 | 0.019 | 0.001 | 0.206 |
| **PC 36:4** | 1680.0 | 71.3 | 2539.7 | 210.2 | 2698.7 | 153.8 | 2686.2 | 160.1 | <0.001 | <0.001 | <0.001 | <0.001 | 0.683 | 0.446 | 0.948 |
| **PC 36:3** | 158442.6 | 6782.5 | 162013.2 | 15007.8 | 173541.5 | 10411.3 | 158761.6 | 9506.6 | 0.388 | * | * | * | * | * | * |
| **PC 36:2** | 3216.2 | 126.3 | 4353.5 | 222.3 | 4468.4 | 191.7 | 3894.5 | 116.7 | <0.001 | <0.001 | <0.001 | 0.001 | 0.536 | 0.018 | 0.011 |
| **PC 38:6** | 166322.5 | 5025.1 | 197412.7 | 11723.8 | 200618.3 | 9329.4 | 196293.0 | 9244.7 | 0.006 | 0.012 | 0.010 | 0.006 | 0.754 | 0.913 | 0.905 |
| **PC 38:5** | 76311.1 | 2895.7 | 102667.2 | 10993.4 | 111718.1 | 8416.6 | 109306.5 | 7652.8 | <0.001 | 0.004 | 0.001 | 0.001 | 0.525 | 0.429 | 0.773 |
| **PC 38:4** | 1219.7 | 46.4 | 2697.7 | 276.6 | 2949.9 | 220.2 | 3061.1 | 258.8 | <0.001 | <0.001 | <0.001 | <0.001 | 0.298 | 0.293 | 0.644 |
| **PC 40:7** | 22908.1 | 1279.0 | 25155.2 | 2610.4 | 27776.6 | 2499.2 | 29947.2 | 2386.0 | 0.019 | 0.316 | 0.086 | 0.016 | 0.243 | 0.092 | 0.332 |
| **PC 40:6** | 58213.5 | 2397.7 | 77391.5 | 4593.0 | 74810.8 | 3657.4 | 79801.1 | 4998.2 | <0.001 | <0.001 | <0.001 | <0.001 | 0.533 | 0.560 | 0.451 |
| **PC 40:5** | 16510.4 | 600.0 | 22936.3 | 2259.8 | 23011.3 | 1489.4 | 23089.2 | 1316.6 | <0.001 | <0.001 | <0.001 | <0.001 | 0.961 | 0.994 | 0.959 |
| **PG 16:1 18:1** | 19.4 | 1.2 | 40.0 | 4.9 | 49.8 | 4.0 | 39.0 | 3.7 | <.001 | 0.002 | <0.001 | 0.002 | 0.085 | 0.853 | 0.137 |
| **PG 16:0 18:1** | 84.9 | 6.6 | 94.1 | 11.6 | 125.1 | 12.7 | 93.9 | 10.3 | 0.038 | 0.789 | 0.035 | 0.524 | 0.034 | 0.989 | 0.081 |
| **PG 18:1 18:1** | 142.8 | 9.1 | 261.4 | 16.1 | 256.5 | 17.3 | 232.6 | 23.0 | <0.001 | <0.001 | <0.001 | 0.001 | 0.843 | 0.477 | 0.337 |
| **PG 18:0 18:1** | 59.7 | 10.9 | 62.3 | 5.9 | 67.8 | 9.1 | 62.0 | 6.5 | 0.806 | * | * | * | * | * | * |
| **PE 32:1** | 1744.4 | 42.4 | 1262.4 | 100.7 | 1235.8 | 82.5 | 1096.3 | 40.7 | <0.001 | <0.001 | <0.001 | <0.001 | 0.740 | 0.108 | 0.089 |
| **PE 32:0** | 2999.2 | 142.0 | 2956.7 | 246.0 | 2910.2 | 266.1 | 2498.6 | 145.6 | 0.168 | * | * | * | * | * | * |
| **PE 34:2** | 415.5 | 19.9 | 370.3 | 33.1 | 376.0 | 28.0 | 281.0 | 18.3 | 0.003 | 0.370 | 0.241 | 0.002 | 0.865 | 0.011 | 0.020 |
| **PE 34:1** | 21.6 | 2.4 | 21.8 | 2.0 | 21.9 | 1.5 | 23.2 | 1.9 | 0.932 | * | * | * | * | * | * |
| **PE 36:5** | 6956.7 | 240.6 | 5621.7 | 245.3 | 5674.1 | 254.2 | 6328.0 | 226.0 | <0.001 | 0.001 | 0.001 | 0.058 | 0.871 | 0.086 | 0.050 |
| **PE 36:4** | 3685.0 | 127.8 | 3468.6 | 241.1 | 3302.0 | 155.1 | 3454.5 | 147.9 | 0.423 | * | * | * | * | * | * |
| **PE 36:3** | 4130.8 | 167.5 | 5243.0 | 295.3 | 5101.2 | 258.2 | 5735.9 | 305.8 | <0.001 | 0.003 | 0.004 | <0.001 | 0.651 | 0.123 | 0.119 |
| **PE 36:2** | 351.6 | 16.0 | 244.6 | 26.8 | 213.3 | 14.8 | 208.5 | 18.1 | <0.001 | <0.001 | <0.001 | <0.001 | 0.133 | 0.194 | 0.816 |
| **PE 36:1** | 145.7 | 10.6 | 71.8 | 9.5 | 75.7 | 6.0 | 58.3 | 4.8 | <0.001 | <0.001 | <0.001 | <0.001 | 0.704 | 0.196 | 0.220 |
| **PE 36:0** | 53.2 | 4.1 | 36.0 | 3.4 | 32.8 | 2.9 | 27.1 | 2.8 | <0.001 | <0.001 | <0.001 | <0.001 | 0.482 | 0.129 | 0.206 |
| **PE 38:6** | 1326.6 | 56.4 | 994.7 | 91.5 | 866.3 | 45.6 | 1056.4 | 49.9 | <0.001 | <0.001 | <0.001 | 0.002 | 0.120 | 0.448 | 0.062 |
| **PE 38:5** | 1683.8 | 79.0 | 1440.0 | 98.0 | 1379.8 | 113.6 | 1588.4 | 65.6 | 0.039 | 0.085 | 0.046 | 0.393 | 0.589 | 0.188 | 0.158 |
| **PE38:4** | 515.5 | 40.6 | 1283.6 | 88.6 | 1329.4 | 83.6 | 1561.5 | 77.4 | <0.001 | <0.001 | <0.001 | <0.001 | 0.558 | 0.003 | 0.005 |
| **PE 38:3** | 2162.8 | 106.2 | 2911.8 | 211.1 | 3033.1 | 153.1 | 3643.0 | 191.1 | <0.001 | <0.001 | <0.001 | <0.001 | 0.527 | 0.002 | 0.003 |
| **PE 38:2** | 7806.5 | 272.5 | 9526.1 | 603.6 | 9013.4 | 308.4 | 9801.0 | 384.5 | 0.003 | 0.007 | 0.028 | 0.003 | 0.333 | 0.602 | 0.300 |
| **PE 38:1** | 35011.9 | 1958.0 | 45698.9 | 1945.9 | 44578.5 | 1836.7 | 49202.0 | 1120.2 | <0.001 | <0.001 | <0.001 | <0.001 | 0.631 | 0.139 | 0.129 |
| **PE 40:7** | 1686.1 | 89.6 | 2585.1 | 143.8 | 2618.6 | 122.2 | 3019.2 | 142.3 | <0.001 | <0.001 | <0.001 | <0.001 | 0.826 | 0.019 | 0.013 |
| **PE 40:6** | 3586.4 | 252.8 | 5677.5 | 335.9 | 5762.2 | 162.5 | 6475.0 | 315.4 | <0.001 | <0.001 | <0.001 | <0.001 | 0.813 | 0.079 | 0.054 |
| **LPC 14:0** | 771.4 | 39.9 | 728.8 | 57.1 | 747.3 | 34.9 | 660.8 | 23.1 | 0.102 | * | * | * | * | * | * |
| **LPC 15:0** | 902.4 | 33.3 | 750.8 | 32.2 | 679.3 | 14.7 | 638.3 | 19.8 | <0.001 | <0.001 | <0.001 | <0.001 | 0.060 | 0.012 | 0.270 |
| **LPC 16:1** | 8449.6 | 556.8 | 4701.1 | 562.3 | 4990.1 | 392.5 | 4515.2 | 224.7 | <0.001 | <0.001 | <0.001 | <0.001 | 0.574 | 0.717 | 0.624 |
| **LPC 16:0** | 186864.3 | 5565.7 | 174527.1 | 10795.2 | 154173.9 | 3772.6 | 150557.3 | 2836.8 | <0.001 | 0.152 | 0.002 | 0.001 | 0.022 | 0.020 | 0.669 |
| **LPC 18:2** | 97703.7 | 4743.4 | 82779.2 | 5569.5 | 84329.9 | 3071.8 | 81521.3 | 1165.0 | 0.019 | 0.026 | 0.019 | 0.027 | 0.776 | 0.818 | 0.862 |
| **LPC 18:1** | 81386.5 | 3853.7 | 57619.7 | 5020.4 | 57234.3 | 3052.1 | 55425.7 | 2477.0 | <0.001 | <0.001 | <0.001 | <0.001 | 0.931 | 0.872 | 0.684 |
| **LPC 18:0** | 59420.6 | 2563.1 | 98574.4 | 8435.1 | 86695.4 | 3779.9 | 89316.4 | 2285.2 | <0.001 | <0.001 | <0.001 | <0.001 | 0.093 | 0.102 | 0.636 |
| **LPC 20:5** | 2005.3 | 156.6 | 1120.6 | 144.2 | 1330.8 | 168.5 | 1698.2 | 151.5 | <0.001 | <0.001 | <0.001 | 0.021 | 0.107 | <0.001 | 0.007 |
| **LPC 20:4** | 29687.3 | 2206.3 | 36354.8 | 4510.6 | 38420.6 | 3184.2 | 40964.3 | 2038.0 | 0.039 | 0.093 | 0.075 | 0.030 | 0.595 | 0.462 | 0.513 |
| **LPC 20:3** | 9624.2 | 789.4 | 7673.8 | 1313.8 | 8201.0 | 877.0 | 8615.9 | 894.3 | 0.305 | * | * | * | * | * | * |
| **LPC 20:2** | 999.2 | 79.5 | 1056.2 | 121.4 | 1104.0 | 79.0 | 1061.5 | 41.3 | 0.736 | * | * | * | * | * | * |
| **LPC 20:1** | 2247.5 | 138.6 | 1682.6 | 193.3 | 1626.8 | 126.6 | 1491.6 | 61.7 | <0.001 | <0.001 | <0.001 | <0.001 | 0.704 | 0.399 | 0.360 |
| **LPC 20:0** | 1790.2 | 74.4 | 2749.1 | 159.6 | 2370.6 | 75.1 | 2035.8 | 147.4 | <0.001 | <0.001 | 0.008 | 0.181 | 0.043 | 0.001 | 0.072 |
