# Supplementary material for: Plasma Lysophosphatidylcholine Levels Are Reduced in Obesity and Type 2 Diabetes
Source: PLoS One. 2012 Jul 25;7(7):e41456. doi: 10.1371/journal.pone.0041456 (PMC3405068; doi:10.1371/journal.pone.0041456)
Supplement: Table S3 — Relationship between plasma LPC levels and HOMA-IR in high fat fed mice. (DOC) [file pone.0041456.s003.doc]

**Table S3. Relationship between plasma LPC levels and HOMA-IR in high fat fed mice.**

| Variable | Coefficient | SE | t-value | P value | 95% confidence interval |
| --- | --- | --- | --- | --- | --- |
| *HOMA-IR* |  |  |  |  |  |
| LPC 14:0 | -1.78 | 1.57 | -1.13 | 0.28 | [-5.21, 1.65] |
| LPC 15:0 | -2.90 | 1.36 | -2.13 | 0.05 | [-5.85, 0.06] |
| LPC 16:1 | -53.69 | 21.41 | -2.51 | 0.03 | [-100.34, -7.04] |
| LPC 16:0 | -77.17 | 62.81 | -1.23 | 0.24 | [-214.01, 59.68] |
| LPC 18:2 | -51.17 | 88.81 | -0.58 | 0.58 | [-244.66, 142.33] |
| LPC 18:1 | -125.88 | 64.75 | -1.94 | 0.08 | [-266.96, 15.20] |
| LPC 18:0 | 42.94 | 28.45 | 1.51 | 0.16 | [-19.05, 104.93] |
| LPC 20:5 | -19.20 | 9.64 | -1.99 | 0.07 | [-40.20, 1.81] |
| LPC 20:4 | 72.36 | 50.45 | 1.43 | 0.18 | [-37.56, 182.27] |
| LPC 20:3 | -41.07 | 32.77 | -1.25 | 0.23 | [-112.47, 30.34] |
| LPC 20:2 | 0.15 | 4.54 | 0.03 | 0.97 | [-9.74, 10.04] |
| LPC 20:1 | -13.09 | 5.58 | -2.35 | 0.04 | [-25.24, -0.95] |
| LPC 20:0 | -7.65 | 3.06 | -2.50 | 0.03 | [-14.31, -0.99] |
| Sum LPC | -720.73 | 333.09 | -2.16 | 0.05 | [-1446.47, 5.00] |
| *HOMA-IR and diet* |  |  |  |  |  |
| *LPC 14:0* |  |  |  |  |  |
| High fat diet | -157.60 | 43.95 | -3.59 | <0.001 | [-254.33, -60.88] |
| HOMA-IR | 1.73 | 1.48 | 1.16 | 0.27 | [-1.54, 4.99] |
| *LPC 15:0* |  |  |  |  |  |
| High fat diet | -152.65 | 31.57 | -4.83 | <0.001 | [-222.15, -83.16] |
| HOMA-IR | 0.50 | 1.07 | 0.47 | 0.65 | [-1.85, 2.85] |
| *LPC 16:1* |  |  |  |  |  |
| High fat diet | -1875.08 | 674.80 | -2.78 | 0.02 | [-3360.30, -389.86] |
| HOMA IR | -11.98 | 22.79 | -0.53 | 0.61 | [-62.13, 38.17] |
| *LPC 16:0* |  |  |  |  |  |
| High fat diet | -5222.75 | 2046.61 | -2.55 | 0.03 | [-9727.30, -718.20] |
| HOMA-IR | 39.02 | 69.11 | 0.56 | 0.58 | [-113.09, 191.12] |
| *LPC 18:1* |  |  |  |  |  |
| High fat diet | -3174.75 | 2484.19 | -1.28 | 0.23 | [-8642.41, 2292.91] |
| HOMA-IR | -55.26 | 83.88 | -0.66 | 0.52 | [-239.88, 129.37] |
| *LPC 18:0* |  |  |  |  |  |
| High fat diet | 1641.38 | 1059.95 | 1.55 | 0.15 | [-691.54, 3974.31] |
| HOMA-IR | 6.43 | 35.79 | 0.18 | 0.86 | [-72.35, 85.20] |
| *LPC 20:5* |  |  |  |  |  |
| High fat diet | -585.59 | 354.86 | -1.65 | 0.13 | [-1366.64, 195.47] |
| HOMA-IR | -6.17 | 11.98 | -0.51 | 0.62 | [-32.54, 20.20] |
| *LPC 20:4* |  |  |  |  |  |
| High fat diet | 4317.80 | 1614.63 | 2.67 | 0.02 | [764.03, 7871.57] |
| HOMA-IR | -23.70 | 54.52 | -0.43 | 0.67 | [-143.70, 96.30] |
| *LPC 20:1* |  |  |  |  |  |
| High fat diet | -361.44 | 201.70 | -1.79 | 0.10 | [-805.37, 82.49] |
| HOMA-IR | -5.05 | 6.81 | -0.74 | 0.47 | [-20.04, 9.94] |
| *LPC 20:0* |  |  |  |  |  |
| High fat diet | -85.18 | 123.03 | -0.69 | 0.50 | [-355.96, 185.61] |
| HOMA-IR | -5.75 | 4.15 | -1.38 | 0.19 | [-14.89, 3.39] |
| *Sum LPC* |  |  |  |  |  |
| High fat diet | -25936.09 | 11242.85 | -2.31 | 0.04 | [-50681.43, -1190.75] |
| HOMA-IR | -143.76 | 379.64 | -0.38 | 0.71 | [-979.33, 691.82] |

Variable regression anlaysis was performed on each LPC species with HOMA-IR. Linear regression was subsequently performed on those species that differed by diet and/or HOMA-IR. Both diet and HOMA-IR were included in the model to determine if HOMA-IR was an independent predictor of LPC levels. SE, standard error.
