# Supplementary material for: Plasma Lysophosphatidylcholine Levels Are Reduced in Obesity and Type 2 Diabetes
Source: PLoS One. 2012 Jul 25;7(7):e41456. doi: 10.1371/journal.pone.0041456 (PMC3405068; doi:10.1371/journal.pone.0041456)
Supplement: Table S4 — Relationship between plasma LPC levels and body composition in high fat fed mice. (DOC) [file pone.0041456.s004.doc]

**Table S4. Relationship between plasma LPC levels and body composition in high fat fed mice.**

| Variable | Coefficient | SE | t-value | P value | 95% confidence interval |
| --- | --- | --- | --- | --- | --- |
| *Body composition* |  |  |  |  |  |
| *LPC 14:0* |  |  |  |  |  |
| % fat mass | -6.56 | 1.55 | -4.22 | <0.001 | [-9.94, -3.17] |
| % lean mass | 6.30 | 1.67 | 3.77 | <0.001 | [2.66, 9.95] |
| *LPC 15:0* |  |  |  |  |  |
| % fat mass | -7.44 | 0.98 | -7.62 | <0.001 | [-9.56, -5.31] |
| % lean mass | 7.31 | 1.10 | 6.64 | <0.001 | [4.91, 9.71] |
| *LPC 16:1* |  |  |  |  |  |
| % fat mass | -96.65 | 27.39 | -3.53 | <0.001 | [-156.32, -36.97] |
| % lean mass | 92.08 | 29.17 | 3.16 | 0.01 | [28.53, 155.63] |
| *LPC 16:0* |  |  |  |  |  |
| % fat mass | -171.02 | 85.31 | -2.00 | 0.07 | [-356.90, 14.86] |
| % lean mass | 169.66 | 86.60 | 1.96 | 0.07 | [-19.02, 358.34] |
| *LPC 18:2* |  |  |  |  |  |
| % fat mass | -142.64 | 126.64 | -1.13 | 0.28 | [-418.55, 133.28] |
| % lean mass | 135.93 | 128.55 | 1.06 | 0.31 | [-144.16, 416.01] |
| *LPC 18:1* |  |  |  |  |  |
| % fat mass | -167.71 | 98.58 | -1.70 | 0.12 | [-382.49, 47.08] |
| % lean mass | 152.07 | 101.79 | 1.49 | 0.16 | [-69.71, 373.85] |
| *LPC 18:0* |  |  |  |  |  |
| % fat mass | 111.83 | 32.63 | 3.43 | 0.01 | [40.73, 182.93] |
| % lean mass | -111.06 | 33.45 | -3.32 | 0.01 | [-183.94, -38.18] |
| *LPC 20:5* |  |  |  |  |  |
| % fat mass | -28.04 | 14.32 | -1.96 | 0.07 | [-59.23, 3.16] |
| % lean mass | 29.13 | 14.31 | 2.03 | 0.07 | [-2.06, 60.31] |
| *LPC 20:4* |  |  |  |  |  |
| % fat mass | 208.73 | 53.77 | 3.88 | <0.001 | [91.58, 325.88] |
| % lean mass | -220.23 | 51.02 | -4.32 | <0.001 | [-331.38, -109.07] |
| *LPC 20:3* |  |  |  |  |  |
| % fat mass | -7.70 | 51.5 | -0.15 | 0.88 | [-119.91, 104.52] |
| % lean mass | 3.69 | 52.02 | 0.07 | 0.95 | [-109.66, 117.03] |
| *LPC 20:2* |  |  |  |  |  |
| % fat mass | 3.61 | 6.63 | 0.54 | 0.60 | [-10.84, 18.07] |
| % lean mass | -4.40 | 6.66 | -0.66 | 0.52 | [-18.91, 10.10] |
| *LPC 20:1* |  |  |  |  |  |
| % fat mass | -20.95 | 7.92 | -2.65 | 0.02 | [-38.20, -3.69] |
| % lean mass | 20.74 | 8.08 | 2.57 | 0.03 | [3.13, 38.35] |
| *LPC 20:0* |  |  |  |  |  |
| % fat mass | -9.30 | 4.89 | -1.90 | 0.08 | [-19.95, 1.35] |
| % lean mass | 9.40 | 4.93 | 1.90 | 0.08 | [-1.35, 20.14] |
| *LPC 22:6* |  |  |  |  |  |
| % fat mass | 94.93 | 44.35 | 2.14 | 0.05 | [-1.69, 191.55] |
| % lean mass | -93.88 | 45.10 | -2.08 | 0.06 | [-192.15, 4.38] |
| *Sum LPC* |  |  |  |  |  |
| % fat mass | -1159.30 | 474.81 | -2.44 | 0.03 | [-2193.78, -124.74] |
| % lean mass | 1130.25 | 487.14 | 2.32 | 0.04 | [68.86, 2191.65] |

Regression analysis was performed on each LPC species with percent fat and lean mass. SE, standard error.
