# Supplementary material for: Plasma Lysophosphatidylcholine Levels Are Reduced in Obesity and Type 2 Diabetes
Source: PLoS One. 2012 Jul 25;7(7):e41456. doi: 10.1371/journal.pone.0041456 (PMC3405068; doi:10.1371/journal.pone.0041456)
Supplement: Table S5 — Relationship between plasma LPC levels, body composition and diet in high fat fed mice. (DOC) [file pone.0041456.s005.doc]

**Table S5. Relationship between plasma LPC levels, body composition and diet in high fat fed mice.**

| Variable | Coefficient | SE | t-value | P value | 95% confidence interval |
| --- | --- | --- | --- | --- | --- |
| *LPC 14:0* |  |  |  |  |  |
| High fat diet | -12.80 | 88.67 | -0.14 | 0.89 | [-207.97, 182.36] |
| % fat mass | -5.96 | 4.43 | -1.35 | 0.21 | [-15.71, 3.78] |
| High fat diet | -56.08 | 94.41 | -0.59 | 0.57 | [-263.88, 151.72] |
| % lean mass | 3.67 | 4.76 | 0.77 | 0.46 | [-6.81, 14.14] |
| *LPC 15:0* |  |  |  |  |  |
| High fat diet | -32.35 | 54.87 | -0.59 | 0.57 | [-153.12, 88.43] |
| % fat mass | -5.93 | 2.74 | -2.17 | 0.05 | [-11.97, 0.10] |
| High fat diet | -58.94 | 60.56 | -0.97 | 0.35 | [-192.24, 74.35] |
| % lean mass | 4.54 | 3.05 | 1.49 | 0.17 | [-2.18, 11.26] |
| *LPC 16:1* |  |  |  |  |  |
| High fat diet | -2298.65 | 1402.67 | -1.64 | 0.13 | [-5385.90, 788.61] |
| % fat mass | 10.19 | 70.06 | 0.15 | 0.89 | [-144.00, 164.38] |
| High fat diet | -3105.82 | 1384.95 | -2.24 | 0.05 | [-6154.08, -57.56] |
| % lean mass | -53.91 | 69.82 | -0.77 | 0.46 | [-207.57, 99.76] |
| *LPC 18:0* |  |  |  |  |  |
| High fat diet | -2364.90 | 1722.29 | -1.37 | 0.20 | [-6155.64, 1425.84] |
| % fat mass | 221.75 | 86.02 | 2.58 | 0.03 | [32.42, 411.08] |
| High fat diet | -2199.09 | 1798.95 | -1.22 | 0.25 | [-6158.54, 1760.36] |
| % lean mass | -214.42 | 90.69 | -2.36 | 0.04 | [-414.03, -14.82] |
| *LPC 20:4* |  |  |  |  |  |
| High fat diet | -250.79 | 3070.57 | -0.08 | 0.94 | [-7010, 6507] |
| % fat mass | 220.39 | 153.36 | 1.44 | 0.18 | [-117.16, 557.93] |
| High fat diet | -1667.00 | 2881.00 | -0.58 | 0.58 | [-8007, 4674] |
| % lean mass | -298.56 | 145.22 | -2.06 | 0.06 | [-618.19, 21.06] |
| *LPC 20:1* |  |  |  |  |  |
| High fat diet | -520.48 | 424.28 | -1.23 | 0.25 | [-1454.32, 413.35] |
| % fat mass | 3.24 | 21.19 | 0.15 | 0.88 | [-43.40, 49.88] |
| High fat diet | -585.05 | 428.26 | -1.37 | 0.20 | [-1527.65, 357.55] |
| % lean mass | -6.76 | 21.59 | -0.31 | 0.76 | [-54.28, 40.76] |
| *Sum LPC* |  |  |  |  |  |
| High fat diet | -53286 | 21851 | -2.44 | 0.03 | [-101382, -5191] |
| % fat mass | 1317.40 | 1091.39 | 1.21 | 0.25 | [-1084.73, 3719.54] |
| High fat diet | -59977 | 21276 | -2.82 | 0.02 | [-106804, -13149] |
| % lean mass | -1688.83 | 1072.54 | -1.57 | 0.14 | [-4049.47, 671.80] |

Diet was subsequently included in the model with those LPC species that showed an association with percent fat or lean mass. SE, standard error.
