# Supplementary material for: Plasma Lysophosphatidylcholine Levels Are Reduced in Obesity and Type 2 Diabetes
Source: PLoS One. 2012 Jul 25;7(7):e41456. doi: 10.1371/journal.pone.0041456 (PMC3405068; doi:10.1371/journal.pone.0041456)
Supplement: Table S6 — Correlations between BMI and circulating insulin levels with plasma LPC species in the human cohort. (DOC) [file pone.0041456.s006.doc]

**Table S6.** Correlations between BMI and circulating insulin levels with plasma LPC species in the human cohort.

| LPC species | BMI | | Plasma insulin | |
| --- | --- | --- | --- | --- |
|  | R value | P value | R value | P value |
| LPC 14:0 | -0.019 | 0.919 | 0.131 | 0.488 |
| LPC 15:0 | -0.397 | 0.029 | -0.157 | 0.406 |
| LPC 16:0 | -0.115 | 0.547 | 0.047 | 0.805 |
| LPC 16:1 | -0.012 | 0.949 | -0.086 | 0.653 |
| LPC 18:0 | -0.555 | 0.001 | -0.278 | 0.137 |
| LPC 18:1 | -0.495 | 0.005 | -0.447 | 0.013 |
| LPC 18:2 | -0.594 | <0.001 | -0.463 | 0.010 |
| LPC 20:0 | -0.553 | 0.001 | -0.494 | 0.006 |
| LPC 20:1 | -0.572 | <0.001 | -0.421 | 0.021 |
| LPC 20:2 | -0.579 | <0.001 | -0.375 | 0.041 |
| LPC 20:3 | -0.345 | 0.062 | -0.324 | 0.081 |
| LPC 20:4 | -0.462 | 0.010 | -0.348 | 0.059 |
| LPC 20:5 | -0.214 | 0.258 | -0.306 | 0.100 |
| LPC 22:6 | -0.281 | 0.132 | -0.465 | 0.010 |
| Sum LPC | -0.441 | 0.015 | -0.340 | 0.066 |
