# Supplementary material for: Plasma Lysophosphatidylcholine Levels Are Reduced in Obesity and Type 2 Diabetes
Source: PLoS One. 2012 Jul 25;7(7):e41456. doi: 10.1371/journal.pone.0041456 (PMC3405068; doi:10.1371/journal.pone.0041456)
Supplement: Table S7 — The fatty acid profile of the low and high fat diet. (DOC) [file pone.0041456.s007.doc]

**Table S7.** Fatty acid composition of the low and high fat diet.

| **Fatty acid** | **LFD** | **HFD** |
| --- | --- | --- |
| 14:0 | 0.7 | 1.3 |
| 16:0 | 11.4 | 24.7 |
| 16:1 | 0.2 | 1.7 |
| 18:0 | 3.2 | 15.7 |
| 18:1 | 43.2 | 32.8 |
| 18:2 n6 | 29.5 | 19.0 |
| 18:3 n3 | 6.8 | 2.1 |
| 20:1 | 0.7 | 0.9 |

Data are percentage of total fatty acid as provided by the manufacturer. LFD, low fat diet; HFD, high fat diet.
